# Constructions of quorum sensing signaling network for activated sludge microbial community

**DOI:** 10.1093/ismeco/ycae018

**Published:** 2024-01-27

**Authors:** Ying Jin, Wenkang Chen, Jie Hu, Jinfeng Wang, Hongqiang Ren

**Affiliations:** State Key Laboratory of Pollution Control and Resource Reuse, School of the Environment, Nanjing University, Nanjing 210023, China; State Key Laboratory of Pollution Control and Resource Reuse, School of the Environment, Nanjing University, Nanjing 210023, China; State Key Laboratory of Pollution Control and Resource Reuse, School of the Environment, Nanjing University, Nanjing 210023, China; State Key Laboratory of Pollution Control and Resource Reuse, School of the Environment, Nanjing University, Nanjing 210023, China; State Key Laboratory of Pollution Control and Resource Reuse, School of the Environment, Nanjing University, Nanjing 210023, China

**Keywords:** activated sludge, quorum sensing, microbial interactions, microbial community, communication network

## Abstract

In wastewater treatment systems, the interactions among various microbes based on chemical signals, namely quorum sensing (QS), play critical roles in influencing microbial structure and function. However, it is challenging to understand the QS-controlled behaviors and the underlying mechanisms in complex microbial communities. In this study, we constructed a QS signaling network, providing insights into the intra- and interspecies interactions of activated sludge microbial communities based on diverse QS signal molecules. Our research underscores the role of diffusible signal factors in both intra- and interspecies communication among activated sludge microorganisms, and signal molecules commonly considered to mediate intraspecies communication may also participate in interspecies communication. QS signaling molecules play an important role as communal resources among the entire microbial group. The communication network within the microbial community is highly redundant, significantly contributing to the stability of natural microbial systems. This work contributes to the establishment of QS signaling network for activated sludge microbial communities, which may complement metabolic exchanges in explaining activated sludge microbial community structure and may help with a variety of future applications, such as making the dynamics and resilience of highly complex ecosystems more predictable.

## Introduction

Quorum sensing (QS) is a cell-to-cell communication process that enables bacteria to collectively modify behavior in response to variations in the cell density and species composition within the surrounding microbial community. QS hinges on the production, release, and group-wide detection of extracellular signaling molecules known as autoinducers [1]. Major classes of these bacterial cell-to-cell signaling molecules encompass N-acyl homoserine lactones (AHLs), autoinducer peptides (AIPs), and autoinducer-2 (AI-2) [2]. Gram-negative bacteria mainly utilize AHLs, a class of autoinducers characterized by a conserved homoserine lactone ring and a hydrophobic acyl side chain, for bacterial communication [3]. Gram-positive bacteria typically employ AIPs, which are linear or cyclic peptides, to facilitate communication among themselves [4]. Interspecies communication, a phenomenon transcending species boundaries, involves numerous Gram-negative and Gram-positive bacteria that participate in QS systems centered around AI-2 [5]. In addition, further studies described other signaling molecules, such as indole [6], diffusible signal factors (DSFs) [7], cholera autoinducer-1 (CAI-1) [8], autoinducer-3 (AI-3) [9], 4-hydroxy-2-alkylquinolines (HAQs) [10], dialkylresorcinols (DARs) [11] and photopyrone [12].

Bacteria monitor changes in the concentration of autoinducers to gauge alterations in their cell numbers [13]. This system allows them to collectively orchestrate comprehensive patterns of gene expression that underlie metabolic activities [14], shaping microbial interactions and the dynamics of microbial communities. The functioning of microbial communities is inextricably associated with various aspects, including substance metabolism, biogeochemical cycles, species evolution, and ecosystem stability. These communities decisively impact processes related to biosynthesis, biodegradation, and wastewater treatment. In the realm of biological wastewater treatment, QS serves as a regulatory system governing multiple behaviors, such as sludge granulation [15], biofilm formation [16], antimicrobial compound synthesis [17], and the secretion of exoenzyme [18]. Researchers have proposed the addition of exogenous AHLs to enhance exopolysaccharide production and promote bacterial aggregation in anammox consortia [19]. Furthermore, dosing exogenous AHLs has been found to facilitate the transition of microorganisms from planktonic growth to sessile growth [20]. Throughout the biofilm development process, QS bacteria exhibit remarkable network centralities, positioning themselves as keystone taxa [21]. The structure and composition of microbial communities can be regulated by QS systems. Exploiting AI-2 as a general communication signal, as opposed to AHLs-like species-specific autoinducers, could be a strategy that enables the host to maximally communicate with and drive global changes in gene expression in mixed populations [22, 23]. Therefore, understanding the relationships between QS systems and microbial communication mechanisms and constructing a QS signaling network for activated sludge microbial communities holds great potential for improving the efficiency of biological wastewater treatment by regulating microbial behaviors.

The construction of a QS signaling network necessitates the establishment of a comprehensive QS database encompassing a wide array of activated sludge microbes and their respective QS systems. Various databases focusing on diverse QS systems and QS-based interactions for different organisms have been developed. For QS signal molecules, repositories of a set of QS signaling molecules and QS signaling peptides in Gram-negative and Gram-positive bacteria have previously been curated to form SigMol [24] and Quorumpeps [25], respectively. Two-component system (TCS) signal transduction proteins have been compiled in P2CS [26]. These databases have been collected separately and lack the network character focusing on signals shared between different species of natural microbiota. Concerning QS-based interactions for different organisms, the QSDB [27] database collected all published sensing and quenching relations between organisms and signaling molecules of the human microbiome, and the QSHGM [28] database visualized and deciphered intricate QS-based interactions for human gut microbiota. Research on the QS-based interactions for different organisms in human microbiome is well-established. However, there is currently no QS database explicitly focusing on the wastewater treatment field. This is because the coexistence and interaction of diverse QS signals in natural microbial communities pose significant challenges in comprehending the types, relative abundances, and QS-controlled behaviors in activated sludge ecosystems.

In this study, we aim to achieve the following objectives: (i) to establish a specific database AS-QSB (activated sludge quorum sensing bacteria) for QS containing abundant entries for activated sludge microbial communities, (ii) to construct and analyze the complex QS signaling network of activated sludge microbial communities for potential mechanisms of intraspecies and interspecies microbial interactions with the help of AS-QSB, and (iii) to complement metabolic exchanges in explaining activated sludge microbial community structure for making the dynamics and resilience of highly complex ecosystems more predictable.

## Materials and methods

### Dataset acquisition

**Figure 1:**
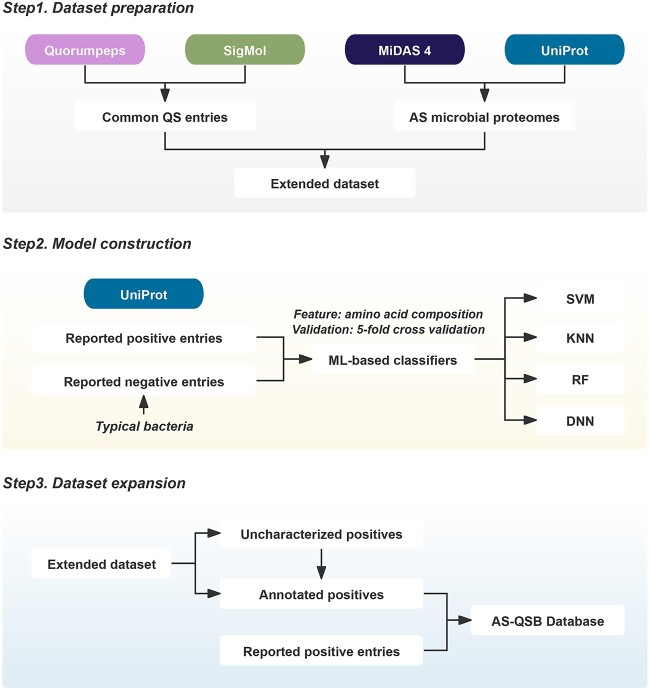
Construction workflow of the AS-QSB database; data from Quorumpeps, SigMol, MiDAS 4, and UniProt were collected as of 30 May 2023.

The entire workflow for AS-QSB database construction has been conducted (Fig. 1). The study utilized auto-inducer synthases and receptors for Gram-negative bacteria from SigMol [24] (https://bioinfo.imtech.res.in/manojk/sigmol/), and QS receptors for Gram-positive bacteria from Quorumpeps [25] (https://quorumpeps.ugent.be/) as common QS entries. The corresponding amino acid sequences were obtained from UniProt [29] (https://www.uniprot.org/). An evolutionary analysis was performed for the validated QS entries using ClustalW algorithms in MEGA 11 [30] for multiple sequence alignment and iTOL [31] (https://itol.embl.de/) for visualization. The evolutionary history was inferred using the Neighbor-Joining method with default options, and the evolutionary distances were computed using the Poisson correction method. The taxonomic dendrogram was drawn to scale, with branch lengths in the same units as those of the evolutionary distances, and was dissected into different QS signal languages distinguished by different backgrounds. The species-level microbes for activated sludge in wastewater treatment plants were obtained from MiDAS 4 [32] (https://www.midasfieldguide.org/guide), and their corresponding proteomes were also obtained from UniProt [29]. An extended dataset was obtained from the results of the local Protein BLAST [33] on common QS-related entries and activated sludge microbial proteomes with the criteria [34] of the E value being smaller than 10^−5^, which is commonly used in sequence alignment to obtain homologs.

The positive samples were manually collected by searching both QS and TCS entries of activated sludge microbes, focusing on commonly used QS annotations such as QS, LuxR, tryptophanase, and two-component. In total 152 551 entries were obtained. Negative samples were generated by excluding protein directly or indirectly associated with QS from proteomes of representative Gram-negative bacteria (*Aliivibrio fischeri*, *Escherichia coli*, *Pseudomonas aeruginosa*, *Salmonella typhimurium*, *and Vibrio parahaemolyticus*) and Gram-positive bacteria (*Bacillus subtilis*, *Staphylococcus aureus*, *and Lactococcus lactis*). The excluded proteins encompassed various functional categories, including QS, luxR, two-component, homoserine-lactone synthase, histidine kinase, biofilm, autoinducer, bacteriocin, competence, virulence, signal, sensor, response, regulator, membrane, binding, transcriptional activator, etc. In total 144 323 entries were obtained.

### Dataset expansion

After excluding the entries that were already collected as reported positive entries from the extended dataset, the remaining entries were classified using the four machine learning (ML)-based classifiers mentioned above. The union of the positives predicted by the efficient classifiers was then divided into uncharacterized positives and annotated positives. The uncharacterized positives were manually re-annotated and sorted with the help of UniProt [29], National Center of Biotechnology Information (NCBI)[35], and Phyre2 [36] databases. Furthermore, we conducted functional analysis by examining their specific annotations, sequence similarity, and domains to determine whether an entry has a QS function or not; if so, whether it is a QS synthase or QS receptor. A combination of manual curation, BLASTP-based expansion, and multiple ML-based classifications helped us obtain as many potential QS entries as possible. Finally, the extended QS entries and the reported QS and TCS entries were combined together to form the AS-QSB database. Following manual classification, 193 306 entries were categorized into two groups, and QS synthases were grouped into eight groups based on different types of QS signal languages.

The activated sludge microbial QS signaling network based on these eight QS signal languages was visualized by Gephi [37]. The hierarchical clustering of eight QS languages found in 372 activated sludge microbial species which possess more than (including) five languages was constructed and classified on their phylum and taxonomy using the common taxonomy tree, which is derived from a diverse array of phylogenetic resources available in the NCBI [35] taxonomy database. The resulting tree was visualized by Interactive Tree Of Life (iTOL) [31], drawn to scale, with branch lengths representing evolutionary distances. The outermost layer heatmap indicates the distribution of QS languages within each cluster, where presence is denoted by blue and absence by grey.

### Co-occurrence network and metabolite utilization analysis

Wu *et al*. [38] have determined core bacterial communities in activated sludge at the global and continent scales based on the abundance and occurrence frequency of operational taxonomic units (OTUs), and they found that taxonomic and phylogenetic composition was significantly different between any two continents. Consequently, we focused on the core community (40 OTUs) in Asia, which exhibited the highest diversity and adopted the same criteria as Wu *et al*. [38] for the determination of the core community in each continent. To identify a core OTU for a specific continent, three measures that are complementary to one another were conducted. That is, its mean relative abundance should be top 0.1%, detected in more than 80% of the samples, and made up the top 80% of the reads in more than 50% of the samples of that continent.

Although there may be potential misconnections between OTUs and microbial species, this widely accepted definition has been employed for reidentifications of stains and enables comparisons with previous studies conducted on other systems.

Network analysis between different microbial species was performed to explore the co-occurrence patterns. Pairwise Spearman’s rank coefficients ($\rho$) were calculated between OTUs within the core community using the R package “WGCNA” [39]. Only robust and significant correlations ($\mid r\mid >0.58,\kern0.5em P<.05$) between OTUs were selected for network construction. The cut-off of the network was determined using the generalized Brody distribution (GBD) based Random Matrix Theory (RMT) method [40]. The curated network was then visualized in Cytoscape [41]. The metabolic capacity of these core microbial species was identified with the help of BacDive standardized bacterial Information database [42] and literature that confirmed the isolation of new species. A set of metabolites (65 metabolites) predicted to mediate core microbial interactions was also identified, and a metabolite utilization relationship and metabolite interchange network (MIN) was established based on this.

## Results

### Reported quorum sensing entries

**Figure 2:**
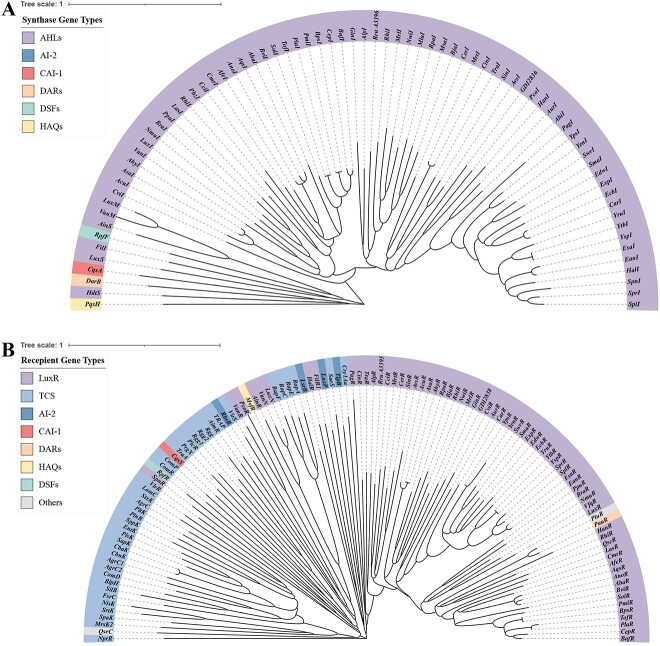
Phylogenetic trees of QS entries from SigMol and Quorumpeps; (A) the optimal tree of QS synthases involves 75 amino acid sequences, and there are a total of 924 sites; (B) the optimal tree of QS receptors involves 117 amino acid sequences, and there are a total of 1026 sites; the color ranges represent different gene types, with the same QS system represented by the same color; it should be noted that photopyrone and AI-3 QS systems are only grouped in recipient genes without corresponding synthase sequences, and TCS has no specific QS system.

Regarding the manual collection results from UniProt database, there are 4811 entries for “Quorum sensing,” 41 341 entries for “LuxR,” 102 884 entries for “Two-component,” and 1421 entries for “Tryptophanase” (Fig. S1). Notably, “Two-component” and “LuxR” account for the majority of entries at 66.95% and 26.90%, respectively. A manual classification was performed to collect information on autoinducer synthase genes and recipient genes according to the SigMol and Quorumpeps databases. The one with the longest sequence was kept while there were identical gene names. The results indicate that 75 autoinducer synthase genes and 77 recipient genes were identified for Gram-negative bacteria, and additionally, 40 autoinducer recipient genes were found for Gram-positive bacteria. Autoinducer synthases were further classified into six different types: AHLs, AI-2, CAI-1, DARs, HAQs, DSFs. Among these types, AHLs synthases were found to be the most abundant (Fig. 2A). QS autoinducer receptors were classified into nine types: LuxR-type, TCS type, AI-2 receptor, CAI-1 receptor, DARs receptor, HAQs receptor, DSFs receptor, photopyrone receptor, and AI-3 receptor. LuxR and TCS type receptors were found to be the most common receptors among QS receptors (Fig. 2B). Only eight kinds of signal molecules were considered in our study, such as AI-3 and photopyrone, whose recipient sequences listed in the database but synthase sequences were unclear, were not included.

### Expanded quorum sensing entries and activated sludge quorum sensing bacteria database

**Figure 3:**
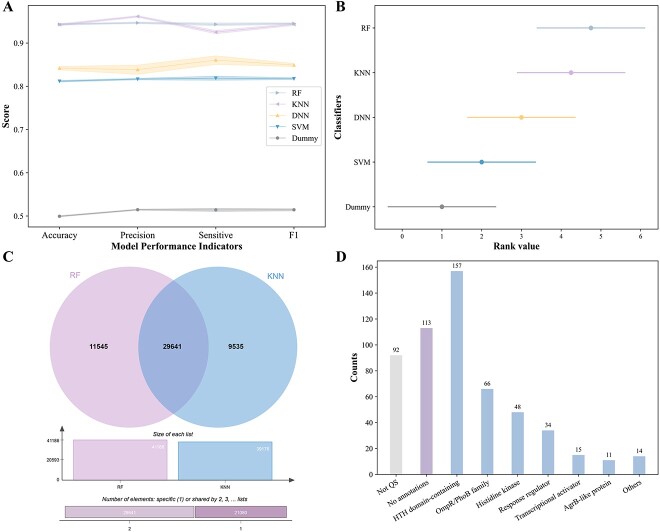
Results of QS entries expansion based on machine learning and functional analysis; (A) accuracy, precision, recall , and F1 score (mean value and confidence intervals of 5-fold cross validation) of the five classifiers based on random classifier (dummy), SVM, DNN, KNN, and RF algorithms (see detailed calculation method in the methods section); (B) Friedman test and post hoc Nemenyi test results of five classifiers; (C) overlaps of the positive results based on two classifiers; (D) results of the protein clusters of 550 re-annotated protein entries; the group “no annotation” means that there are no specific annotations for these entries in UniProt which should be re-annotated manually later.

We calculated the frequency of each amino acid type in each entry sequence as protein features using the amino acid composition (AAC) method. The AAC method calculates the percentage of each amino acid type by dividing the number of each amino acid type by the length of a protein sequence. We conducted a 5-fold cross-validation to train four ML-based classifiers (SVM, DNN, KNN, and RF) and a random classifier (Dummy) with reported positive and negative samples. The average accuracy, prediction, recall, and F1 score were used to evaluate their performances (Fig. 3A). The Friedman test and post hoc Nemenyi results show that the k-nearest neighbor (KNN) and random forest (RF) classifiers exhibit a statistically significant difference when compared to the Random Classifier, while the support vector machine (SVM) and deep neural network (DNN) classifiers do not (Fig. 3B). Given these findings, we have chosen to utilize the combined prediction results of the KNN and RF classifiers, suggesting that the combined predictions from these classifiers may offer a more robust and reliable outcome. We then manually checked the annotations of the predicted results from the classification of the two ML-based classifiers and divided the positives into annotated positives and uncharacterized positives. These were analyzed further for their specific overlaps (Fig. 3C). To obtain as many potential QS entries as possible, it was helpful to combine the four positives from the four classifiers together to form a union. With the help of family and domain information, we re-annotated 550 uncharacterized entries and manually grouped them into nine protein clusters (Fig. 3D) and 345 entries have been re-annotated based on the functional analysis (Table S1). Among these clusters, the helix-turn-helix (HTH) domain-containing protein (a transcriptional activator for QS control of luminescence) occupied the majority. OmpR/PhoB family, histidine kinase, response regulator, transcriptional activator, and AgrB-like protein are followed in turn. There were 92 entries re-annotated to be without QS function. Note that there were another 113 entries that were vaguely described without family and domain information. These entries were further explored and re-annotated based on Phyre2 [36]. There were nine entries (Table 1) that have confident homology templates. A0A1H0L543, A0A6C2YR07, A0A4V1KJR0, E4T6T3 are templated on the DNA-binding protein that mediates transcription activation, while W1HNE0 is templated on the tetratricopeptide repeat-containing protein that mediates signal transduction. A0A6B8RFK9, G5KCT1 are templated on the response regulator that is closely related to the transcriptional regulation. A0A5C0B320 and A0A2T7H6Q3 are templated on the histidine kinase and connector protein in a TCS, respectively. There was a 30.53% increase in extended entries from the previous annotation-based collections.

**Table 1:** Results of another 10 expanded entries based on Phyre2.

**Strain**	**Tax ID**	**Entry**	**Template**	**Confidence**	**Coverage**	**New annotations**
*Prevotella* sp*.*	645 273	A0A1H0L543	d2frha1	91%	27%	Putative DNA-binding MarR family transcriptional regulator
*Paludibacter propionicigenes*	694 427	E4T6T3	c3cloC_	98.20%	93%	Putative LuxR like DNA-binding transcriptional regulator
*Hansschlegelia zhihuaiae*	405 005	A0A4V1KJR0	c1rr7A_	99.50%	68%	Putative middle operon repressor
*Tuwongella immobilis*	692 036	A0A6C2YR07	c3mnlA_	94.20%	94%	Putative TetR family transcriptional regulator
*Klebsiella pneumoniae*	1 432 561	W1HNE0	c6mfvC_	99.80%	81%	Putative tetratricopeptide repeat-containing protein
*Streptococcus urinalis*	764 291	G5KCT1	c4cbvE_	96%	92%	Putative ComE response regulator of the competence regulon
*Paenibacillus psychroresistens*	1 778 678	A0A6B8RFK9	c2p7jA_	98.30%	63%	Putative putative sensory box/ggdef family protein
*Pigmentiphaga aceris*	1 940 612	A0A5C0B320	c5jefA_	99.70%	82%	Putative nitrate/nitrite sensor histidine kinase NarQ
*Labrenzia* sp*.*	2 171 494	A0A2T7H6Q3	c8ck1E_	100%	96%	Putative two-component-system connector protein

The AS-QSB (activated sludge QS bacteria) database is made up of the reported and extended entries related to QS functional proteins mentioned above and is publicly available at http://www.njuqsb.com with the current version of AS-QSB containing a total of 193 306 QS entries. In the AS-QSB database, we described all protein entries with QS functions. Specifically, 14 450 entries are clustered to QS synthases, while the remaining 178 856 entries are clustered to QS receptors based on protein annotations and functional analysis. The synthases were then classified into eight subgroups of QS systems. The HAQs system contained the maximum number of synthetic entries (3359), followed by DSFs (3096), CAI-1 (2376), AHLs (2006), indole (1312), AI-2 (1180), and DARs (1110). The AIPs system contained the least number of QS synthetic entries (11). AIPs consist of a short sequence without a fixed structure, which may lead to an increase in false positives in the BLAST process and inaccuracies in the machine learning process. Therefore, AIPs are not considered in the classification process in our study. The AS-QSB is built from 4228 identified microbial species from MiDAS 4 database, and 3878 (91.72%) of them are QSB with QS functions, which indicates that QS is a ubiquitous and important microbial communication mode in activated sludge microbial communities. The construction of a publicly available QS database for activated sludge microbes provides a reference for the search of signal molecules, especially in less-studied microorganisms.

### Quorum sensing communication network constructions

**Figure 4:**
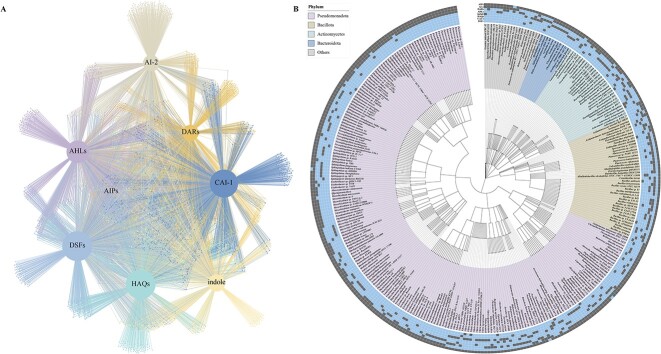
QS communication network for activated sludge microbes based on diverse QS languages; (A) QS communication network for 3787 activated sludge QS bacteria based on eight QS systems (AHLs, DSFs, HAQs, CAI-1, AIPs, DARs, indole, and AI-2); there are two types of nodes, that is QS languages and QS bacteria, and the correlation between them means the specific bacteria have the potential to utilize corresponding QS system; (B) hierarchical clustering of eight QS languages found in 322 activated sludge microbial species which has the potential to utilize more than five QS languages; the constructions are classified into five phylum-level clusters (*Pseudomonadota*, *Bacillus*, *Actinomycetota*, *Bacteroidota*, and others) based on their phyla and taxonomy; the heatmap on the outermost layer indicates QS language distribution in each cluster, with existence marked as present or absent.

Microbes communicate via various QS signals (also termed as microbial languages), and it is possible to construct a cell–cell communication network among different activated sludge microbes based on diverse QS languages, which we termed as QS networks. Based on a review of previous studies, we focused on the common eight QS languages, i.e. AHLs, DSFs, HAQs, CAI-1, AIPs, DARs, indole, and AI-2, to construct the proposed QS network. We assume that strains that can produce a signaling molecule can also receive that signaling molecule. With the help of the AS-QSB database and the hypotheses, we constructed an undirected and bipartite network involving two types of nodes, namely QS signals and microbes for the 3878 activated sludge QSB based on the above eight QS languages (Fig. 4A). Although some strains do not have the function of producing QS signal molecules, they can receive QS signal molecules, which is not shown in our QS network. This intricate network visualizes complex QS-based communications among activated sludge microbes. Different microbes are linked through various languages to form a microbial communication network, and connections could be used to regulate microbial interactions between themselves and the surrounding ones. Most strains produce DSFs (1936, 46.46% of activated sludge microbes) as the communication language, followed by HAQs (1905, 45.72%), CAI-1 (1808, 43.39%), AHLs (1451, 34.82%), indole (1089, 26.13%), AI-2 (1027, 24.65%), DARs (1011, 24.26%), and AIPs (9, 0.22%).

We conducted taxonomy distribution of QS languages at the family level (Fig. S2). The majority of entries belong to *Pseudomonadota*, *Bacillus*, *Actinomycetota*, and *Bacteroidota* at the phylum level, and *Pseudomonadota* bacteria contribute a large proportion of sequences to the QSP database. We also conducted hierarchical clustering of eight QS languages found in 322 activated sludge microbes at the genus level, which produce more than five kinds of QS languages (Fig. 4B). *Pseudomonadota* bacteria have the potential to communicate with almost all QS systems that indicate complex interactions occurring within the bacterial domain. Certain genus from the *Bacillus*, *Actinomycetota*, and *Bacteroidota* phyla have several QS communication systems. It is worth noting that although each microbe has the potential to communicate using multiple QS languages (Fig. 4B), the specific intensity for each language cannot be determined in this study. This research aims to establish the existence of QS-based communications, which can be useful for studying the potential behavior of microorganisms and their interactions with other species in activated sludge. Upset plots for different QS language sets interactions (except for AIPs) at the genus level (Fig. S3A) and at the species level (Fig. S3B) were conducted for activated sludge microbes.

### Co-occurrence network and metabolite exchange network constructions

**Figure 5:**
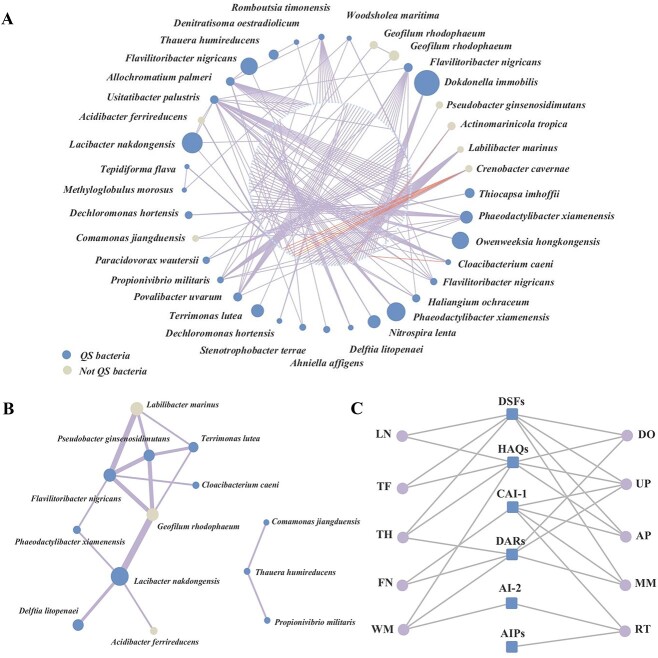
Comparison of co-occurrence network, metabolite exchange network, and partial QS communication network for continent-level core microbial communities; (A) co-occurrence network for core microbial communities in Asia, and only those correlated to core communities were visualized; out layer nodes represent core communities, and inner layer represents those related to core communities; node properties indicate whether the microbe is QS bacteria or not while node size represents the mean relative abundance of these microbes in global samples; (B) metabolite exchange network for activated sludge core microbial communities in Asia; the correlation between them means that they share at least two metabolites; width of lines represents the relative number of metabolites they have in common; node properties follow the same convention as in (A); (C) bipartite QS communication network for 10 selected activated sludge core microbial communities in Asia.

Microbial communities and their functions are shaped by both metabolic interactions and communication-based regulations [43]. On the other hand, a QS-based network and a metabolite exchange network can be co-present and function collectively in a microbial ecosystem. To illustrate the potential of complementary use of the QS network and a metabolic network constructed in this work for the activated sludge microbial community, here we considered the work of Wu *et al*. on the continent-scale core bacterial communities [38]. Core bacterial communities were identified using the same definition as Wu *et al*. (Table S2), and the optimal tree of them was visualized (Fig. S4). We constructed a co-occurrence network for activated sludge microbial communities in Asia (only those related to core species were visualized). MIN for core microbial communities was visualized in chore diagram (Fig. S5), and a metabolite exchange network for core bacterial communities was then conducted (Fig. 5A). As the definition of co-occurrence network, the potential interaction between the two genera of bacteria was defined when forming a co-occurrence pattern.

Most co-occurrence relationships are positive correlations, while *Actinomarinicola tropica* and *Crenobacter cavernae* are mainly negatively associated with other species. Although these core communities are all abundant and widespread species, they are not closely related to each other, while only 11 of them show connection to other core species. *Thauera humireducens*, *Denitratisoma oestradiolicum*, *Romboutsia timonensis*, *Flavilitoribacter nigricans*, *Allochromatium palmeri*, *Usitatibacter palustris*, *Acidibacter ferrireducens*, *Lacibacter nakdongensis*, *Tepidiforma flava*, *Methyloglobulus morosus*, *Woodsholea maritima* were indicated by the acronyms of TH, DO, RT, FN, AP, UP, AF, LN, TF, MM, WM, respectively, and co-occurrence relationships between them included TH-DO, RT-AP, AP-FN, LN-AF, UP-AF, UP-LN, UP-FN, MM-WM, and MM-TF.

Upon comparing the co-occurrence network (Fig. 5A) with the metabolite exchange network (Fig. 5B), it was observed that only the co-occurrence relationship of AF-LN could be elucidated by the metabolite exchange. In contrast, the remaining core intermicrobial connections exhibited no discernible correlation with the exchange of metabolites. Instead, it is conceivable that these links may be mediated by signaling molecules. Applying our AS-QSB database to the Asia core community, we obtained a partial bipartite QS network (Fig. 5C), which shows specific QS-based communications, offering plausible mechanisms for links between core microbial communities that the metabolite exchange network could not explain. It should be noted that all these core microbes that communicate with each other are QS bacteria from the AS-QSB database except for AF, whose proteome does not exist in UniProt database at present. Therefore, we consider our AS-QSB database as a tool that can facilitate the identification of possible QS-based inter-microbial interactions, which may complement metabolic exchanges in a complex community in explaining an observed community structure.

## Discussion

According to recent studies, almost all bacteria in the SNAP reactor microbial community have the ability to produce and release DSF, and the interspecies communication in the bacterial community is mainly mediated by DSF [44]. Our study concluded from global activated sludge samples that most strains (encompassing 46.76% of the species) are observed to both produce and receive DSFs, thereby establishing a prominent communication pattern within activated sludge microbial communities. This phenomenon may be attributed to the versatile nature of DSFs, capable of functioning as both intraspecific and interspecific QS signaling systems, distinguished by their mechanisms from other QS systems. DSF is known to induce biofilm dispersal [45] and to inhibit biofilm development by suppressing the synthesis of extracellular matrix synthesis [46]. It is important to note that the DSF-based QS is widely acknowledged as a conserved mechanism for interspecies communication among Gram-negative bacteria [47]. For instance, DSF family signals facilitate interspecies communication between *Stenotrophomonas maltophilia* and *Burkholderia cenocepacia*. DSFs produced by *S. maltophilia* and *B. cenocepacia* also influence behaviors of other species such as *P. aeruginosa* and *Candida albicans* [48]. Additionally, DSF analogs produced by *P. aeruginosa* also affect the dispersal of mature biofilms and inhibit the development of new biofilm in various species, including *E. coli*, *Klebsiella pneumoniae*, *Proteus mirabilis*, *Streptococcus pyogenes*, *B. subtilis*, *S. aureus*, and *C. albican*. [49].

Previous studies have consistently highlighted the pivotal role of AI-2 and DSFs as the universal language of bacterial communication in interspecific interactions. For instance, AI-2 serves as an interspecies signaling molecule influencing the formation of dual-species biofilms of *Actinomyces naeslundii* and *Streptococcus oralis* [50], and AI-2 secreted by *Porphyromonas gingivalis* slightly stimulates *Fusobacterium nucleatum* biofilm formation [51]. AI-2 QS system has been applied to activated sludge, biofilm, granular sludge, biological denitrification, and other wastewater treatment by promoting extracellular polymeric substances production, regulating community structure, and enhancing bacterial activities [52-54]. Our network analysis suggests the potential involvement of AHLs, indole, CAI-1, HAQs, and DARs in interspecific communication. AHLs QS system is thought to be associated with intraspecific bacterial communication traditionally, with the LuxR protein of each species only recognizing specific AHL signaling molecules, especially AHL released by itself [55]. However, almost all species have AHLs sensors [3] and lactases (AHLs degrading enzymes) [56], even in the absence of AHLs synthetases. CAI-1 is commonly considered to function mainly as an intra-genus communication signal, mainly found in *Vibrio* species [8]. Surprisingly, elevated CAI-1 concentrations can enhance *E. coli* virulence, despite the absence of reported CAI-1 synthase or receptors in *E. coli*, which enables *E. coli* to successfully coordinate host colonization in co-infections with *Vibrio cholerae* [57] and confirms the possibility of CAI-1 involvement in interspecies communication. Bacteria that do not produce indole have developed intrinsic mechanisms to resist indole signaling, facilitating coexistence in multispecies consortia. For example, *P. fluorescens* oxidizes indole to control biofilm formation in *E. coli* within dual-species cultures [58]. HAQs were thought to be produced only by *P. aeruginosa* a few *Burkholderia* species [59], among which PQS signaling is specific to *P. aeruginosa* [60]. Recent studies indicate the potential for HAQs to mediate interspecies communication, and for example, HAQs produced by *P. aeruginosa* are responsible for inhibiting the growth of *S. aureus* under aerobic conditions [61]. Although direct evidence of DARs mediating interspecific communication is lacking, the widespread presence of DAR biosynthetic gene clusters across different bacterial taxa suggests their potential role [62].

We constructed a QS signaling network within the activated sludge microbial community by exploring QS bacteria and their communication systems. Our network comprehensively depicts the QS system of activated sludge microbial community from the perspective of proteomes. Multiple bacterial communication pathways enrich the mechanisms by which bacteria regulate community behavior. For intraspecies communication, pathways mediated by different signal molecules constitute QS network in one species collectively, where multi-channel QS pathways cooperate in different architectures to regulate gene expression. For example, *V. Havii* employs parallel bacterial communication circuits utilizing AHLs, CAI-1, and AI-2 as signal molecules to facilitate intraspecies and interspecies communication [63], controlling biofilm formation and bioluminescence [64]. *P. aeruginosa* employs a hierarchical bacterial communication circuit with signaling molecules comprising two AHLs and two HAQs to enhance bacterial adhesion, biofilm formation, and virulence factor expression [10]. Compared with intraspecies communication, interspecies communication can expand the participation of diverse microorganisms in community succession. It fosters a range of pairwise interactions, including neutral relationships, commensalism, amensalism, predation, cooperation, and competition, all mediated by various QS signals [65]. This Interspecies QS modulation directly influences bacterial physiology by regulating the expression of pertinent genes, thereby shaping the overall community structure [66].

Some prior studies have focused on the ecological interactions of simple marine microbial communities growing on the polysaccharide alginate, where it has been shown that ecological interactions orchestrated by non-degrading community members significantly influence the activities of microbial communities involved in the natural remineralization of carbon biopolymers [67]. We found similar misattribution of metabolite exchange to ecological interactions in activated sludge. Comparing the co-occurrence network with the metabolic interaction network, the connections between core microorganisms are not primarily linked to the exchange of metabolites; instead, these relationships can be attributed to the mediation of signaling molecules. These possible interactions against microbe-QS signal pairs suggested by the database can be validated through experimental approaches, such as detecting and manipulating the production and reception of specific QS signal molecules. However, specific QS molecules used by these core bacterial communities in activated sludge are not extensively characterized in the available scientific literature. It is often difficult to directly couple QS organisms with their putative functions [68] because activated sludge communities are highly diverse, commonly comprised of members from *Proteobacteria*, *Bacteroidetes*, and *Chloroflexi* [69], and often many of those taxa are uncultivable or outcompeted by faster-growing heterotrophs [70], making it challenging to unravel their genetic and physiological attributes and identify individual QS producers and responders. These potential relationships can be verified by KEGG QS pathways in related bacteria. For example, in our activated sludge core microbial co-occurrence network, *T. humireducens*, *D. oestradiolicum*, *U. palustris*, *T. flava*, *Lacibacter* sp*. S13–6-22*, and *Allochromatium vinosum* possess synthetic genes related to PQS and DSF (KEGG ID: thu02024, doe02024, upl02024, tfla02024, lacs02024, alv02024), and it should be noted that *L. nakdongensis* and *A. palmeri* were characterized by the species belonging to the same genus.

Our study also underscores that bacteria often use multiple QS systems to regulate the same cooperative behaviors, that is, the structure of microbial community communication network is redundant. This redundancy in the structure of microbial community communication networks is an outcome of evolution and natural selection, and allows for multiple pathways, components, or receptors to serve as backup mechanisms, ensuring that the network functions effectively in various environments and to prevent interference from natural molecules [71]. This redundancy also allows for specialized functions within the network, allowing bacteria to fine-tune their responses and effectively adapt to complex and dynamic environments [72], thereby contributing to the stability of natural microbial systems [73]. In this context, QS signaling molecules serve as communal resources shared by the entire microbial group, influencing cooperation dynamics [74] and stabilizing QS systems in natural environments [75]. For instance, AHLs, AI-2, DSF, and c-di-GMP can regulate diverse microorganisms with different functions in anaerobic granular sludge (AnGS) simultaneously, including hydrolytic fermenters, acid-producing bacteria, methanogens, and other microorganisms [76]. Recent studies suggest that these signal molecules achieve these effects by selectively enhancing the abundance of microbes related to self-secretion and regulation during the AnGS granulation process [77].

In summary, this research comprehensively depicts the redundant QS system of activated sludge microbial community by constructing a QS signaling network within the activated sludge microbial community. Our results suggest that the connections between core microorganisms in activated sludge microbial communities are primarily linked to the mediation of signaling molecules. Our findings convey the potential involvement of AHLs, indole, CAI-1, HAQs, and DARs in interspecific communication, while DSFs establish a prominent communication pattern. A key aspect of this future work could be to identify the strengths of diverse QS-based interactions, making the dynamics and resilience of highly complex ecosystems more predictable.

## Conflicts of interest

None declared.

## Funding

This work was supported by the Basic Science Center Project of the Natural Science Foundation of China (52388101), National Natural Science Foundation of China (52270041, 52192684, and 51908275), the National Key Research and Development Program of China (2022YFC2105204, 2023YFC3206903), the Jiangsu Provincial Department of Science and Technology (BE2023687), and the Excellent Research Program of Nanjing University (ZYJH005).

## Data availability

About 193 306 redundancy removal entries for activated sludge microbes generated in this study have been deposited in our AS-QSB database which is freely available at: http://www.njuqsb.com. More details for the relevant data of QS entries from Gram-positive microbes, QS entries from Gram-negative microbes, activated sludge microbes, and their corresponding proteomes can be searched in Quorumpeps [25], SigMol [24], MiDAS 4 [32], and UniProt [29] databases, respectively.

## Code availability

The codes for constructing classifiers and data analysis have been provided in a GitHub repository at: https://github.com/jinyingjinying/AS-QSB.

## Supplementary Material

Supporting_information_ycae018
