# Supplementary material for: Constructions of quorum sensing signaling network for activated sludge microbial community
Source: ISME Commun. 2024 Jan 27;4(1):ycae018. doi: 10.1093/ismeco/ycae018 (PMC10945367; doi:10.1093/ismeco/ycae018)
Supplement: Supporting_information_ycae018 [file supporting_information_ycae018.docx]

# Supporting information for

**Constructions of Quorum Sensing Signaling Network for Activated Sludge Microbial Community**

## Models Construction

The protein features were determined by calculating the frequency of each amino acid type in every QS-related protein sequence. In this study, the iFeature package [[1](#_ENREF_1)] was utilized to compute the amino acid information within the protein sequences. The frequencies of all 20 natural amino acids were calculated as a percentage by dividing the number of occurrences by the length of each protein sequence, which is listed as follows:

$$\begin{aligned} f\left( t \right)=\frac{N\left( t \right)}{N}, t \in\left\{ A, C, D,\ldots,Y \right\}\#\left( 1 \right) \end{aligned}$$

where $N\left( t \right)$ is the number of amino acid type $t$, while $N$ is the length of a protein or peptide sequence.

The machine learning algorithms (SVM [[2](#_ENREF_2)], KNN [[3](#_ENREF_3)] and RF [[4](#_ENREF_4)]), and deep learning algorithm (DNN [[5](#_ENREF_5)]) were trained and validated using a fivefold cross-validation approach on the reported positive and negative samples to develop different classifiers. The fivefold cross-validation involved dividing the training set into z parts, where z-1 parts were utilized for training while the remaining part was reserved for validation. All four classifiers were employed to predict whether the input amino acid sequences corresponded to QS entries or not, with the output being 1 (indicating yes) or 0 (indicating no), respectively. The Support Vector Machine (SVM [[2](#_ENREF_2)]), K-nearest-neighbor (KNN [[3](#_ENREF_3)]), and Random forest (RF [[4](#_ENREF_4)]) models were implemented using the sklearn library [[6](#_ENREF_6)] in Python 3.8. In this study, for our results, we employed the GridSearchCv function sklearn [[6](#_ENREF_6)] to automatically select and determine the optimal combition of hyperparameters for achieving superior performance. For SVM [[2](#_ENREF_2)], we utilized the radial basis function (RBF) with standard deviation $\sigma=0.125$ and set the regularization parameter $C=1$ to train both positive and negative samples. Regarding KNN [[3](#_ENREF_3)], we assigned weights proportional to the inverse of the distance from the query point and set n_neighbors $k=5$ which yielded optimal result. As for RF [[4](#_ENREF_4)], we applied max_features as auto and ensured result reproducibility by setting random_seed accordingly. The n_estimator was set to 10 since even a smaller value can deliver excellent performance while saving computation costs. The deep neural network (DNN [[5](#_ENREF_5)]) was implemented using the PyTorch library in Python 3.8. Following testing, the four-layer network with ReLu [[7](#_ENREF_7)] as the activation function between layers exhibited exceptional predictive performance for identifying QS entries. The input layer consists of a specific number of neurons corresponding to input features. In the first layer (one-to-one layer), an equal number of neurons were employed, each connected to one neuron from the input layer. Subsequently two hidden layers were added after the one-to-one layer: the first hidden layer was fully connected to the one-to-one layer and the second hidden layer was fully connected to the first hidden layer. The final output layer comprised only two neurons. A fixed global seed was set for random number generation to ensure reproducible traing results. Stochastic gradient descent (SGD) optimizer was utilized for training the DNN [[5](#_ENREF_5)] model, with fixed values of 30 epochs, a batch size of 64, and a learning rate of 0.1, respectively specified. Default hyper-parameter values were used for other aspects of DNN [[5](#_ENREF_5)] modeling without tuning.

## Models Evaluation

In the domain of machine learning, the assessment of multiple classifiers across diverse datasets is crucial for understanding their relative performances. Performances of the four ML-based classifiers were measured through an comprehensive methodology, which ensures a thorough evaluation of multiple classifiers across varied datasets, providing nuanced insights into their comparative performances. Firstly, the average accuracy, precision, recall, and F1 score of 5-fold cross validation were calculated and they are defined as follows:

$$\begin{aligned} Accuracy=\frac{TN+TP}{TN+TP+FN+FP}\#\left( 2 \right) \end{aligned}$$

$$\begin{aligned} Precision=\frac{TP}{TP+FP}\#\left( 3 \right) \end{aligned}$$

$$\begin{aligned} Recall=\frac{TP}{TP+FN}\#\left( 4 \right) \end{aligned}$$

$$\begin{aligned} F1=2*Precision*\frac{Recall}{Precision+Recall}\#\left( 5 \right) \end{aligned}$$

where $TP$ represents true positives, $TN$ denotes true negatives, $FP$ and $FN$ are false positives and false negatives, respectively. $F1$ score is the harmonic mean of prediction and recall. The higher the $F1$ score is, the better performance the classifier will be of.

Secondly, the Friedman test, a non-parametric method, serves as a robust tool for comparing the performances of three or more learning algorithms on different datasets. Initially, the performance scores of each learner are ranked within each dataset independently. The Friedman test statistic ($\chi_{F}^{2}$) is computed using the formula:

$$\begin{aligned} \chi_{F}^{2}=\frac{12N}{k\left( k+1 \right)}\left[ \sum_{j=1}^{k} R_{j}^{2}-\frac{{k\left( k+1 \right)}^{2}}{4} \right]\#\left( 6 \right) \end{aligned}$$

Here, $N$ represents the number of datasets, $k$ is the number of classifiers, and $R_{j}$ denotes the sum of ranks for the$j$-th learner. The resulting test statistic follows a chi-square distribution with $k$-1 degrees of freedom. Upon rejecting the null hypothesis, suggesting significant differences among the classifiers, post-hoc tests are imperative. The Post-hoc Nemenyi test is a valuable follow-up method, and the critical difference ($CD$) is calculated as:

$$\begin{aligned} q_{\alpha}\times\sqrt{\frac{k\left( k+1 \right)}{6N}}\#\left( 7 \right) \end{aligned}$$

where $q_{\alpha}$ represents the critical value associated with the chosen significance level.

Pairwise comparisons involving the mean ranks of classifiers are then conducted, and if the absolute difference in mean ranks exceeds $CD$, the classifiers are deemed significantly different. Finally, Mann-Whitney U test was used to verify the difference between each two samples with differences in Nemenyi test.

## Fig. S1 Total Quoum Sensing (QS) & Two-component system (TCS) entries with four protein annotations, i.e., “Quorum Sensing”, “LuxR”, “Two-component”, and “Tryptophanase”.

## Fig. S2 Taxonomy distribution of QS languages at the family level. Outlayer heatmap is shown in log10(N+1), while N presents the number of species that communicate with certain QS languages in this family.

**
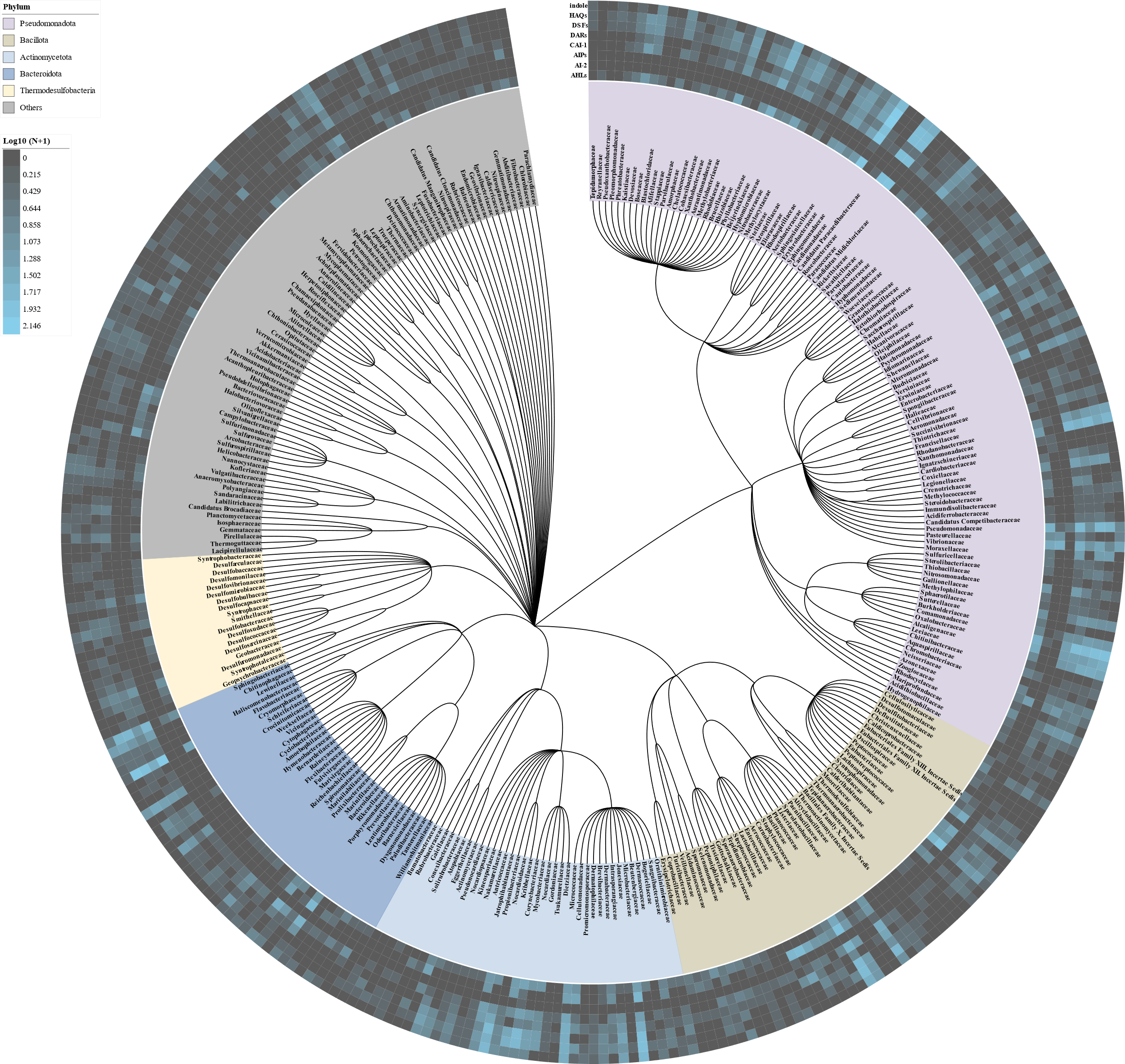
**

## Fig. S3 Upset plots for different QS language sets interactions at the genus level and at the species level.


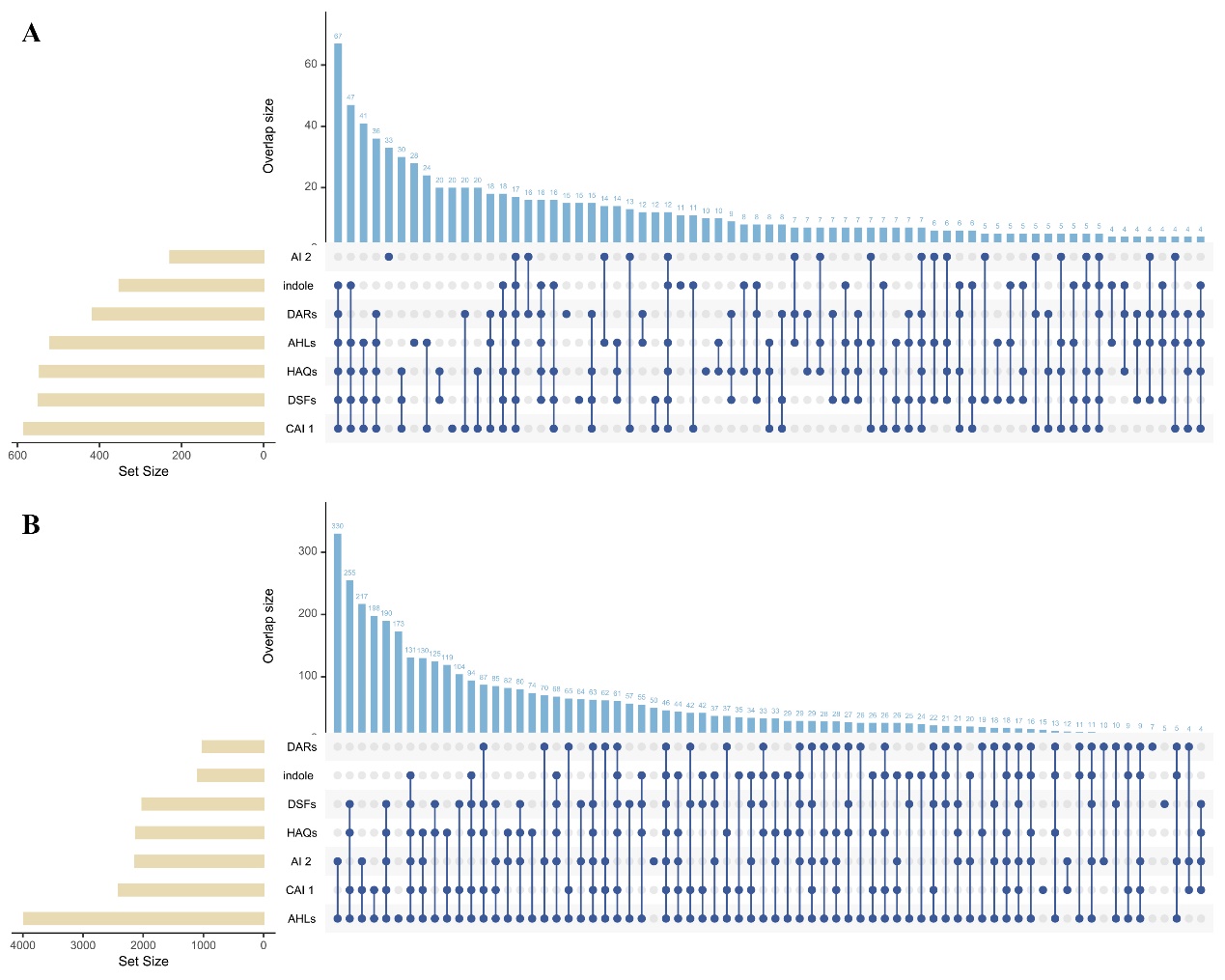


## Fig. S4 Optimal tree of core bacterial communities in Asia activated sludge samples.


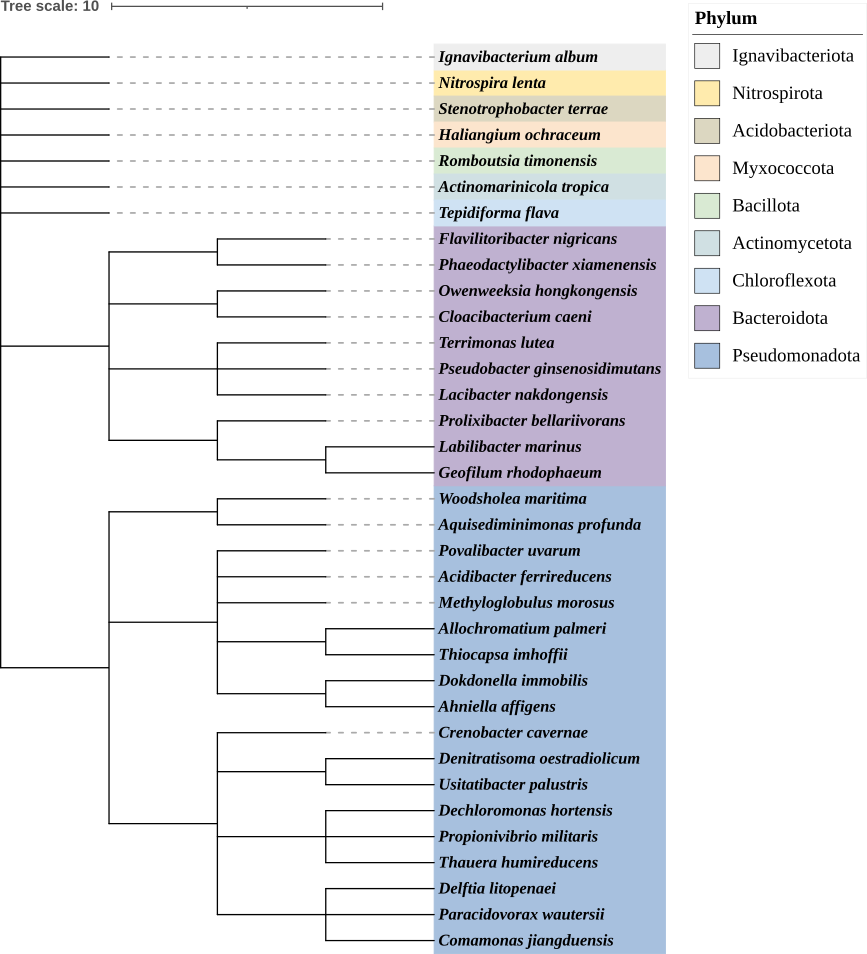


## Fig. S5 Metabolite interchange network for core microbial communities in Asia-activated sludge samples. Crenobacter cavernae, Delftia litopenaei, *Denitratisoma oestradiolicum*, Comamonas jiangduensis, Propionivibrio militaris, *Thauera humireducens*, *Thiocapsa imhoffii*, *Geofilum rhodophaeum*, *Labilibacter marinus*, *Methyloglobulus morosus*, *Flavilitoribacter nigricans*, *Lacibacter nakdongensis*, *Paracidovorax wautersii*, *Tepidiforma flava*, *Phaeodactylibacter xiamenensis*, *Acidibacter ferrireducens*, *Usitatibacter palustris*, *Owenweeksia hongkongensis*, *Terrimonas lutea*, *Povalibacter uvarum*, *Romboutsia timonensis*, *Cloacibacterium caeni*, *Pseudobacter ginsenosidimutans*, *Dokdonella immobilis*, *Dechloromonas hortensis*, *Stenotrophobacter terrae*, *Allochromatium palmeri*, *Actinomarinicola tropica*, *Nitrospira lenta* were indicated by acronyms of CC, DL, DO, CJ, PM, TH, TI, GR, LM, MM, FN, LN, PW, TL, PX, AF, UP, OH, TL, PU, RT, OC, PG, DI, DH, ST, AP, AT, NL.

**
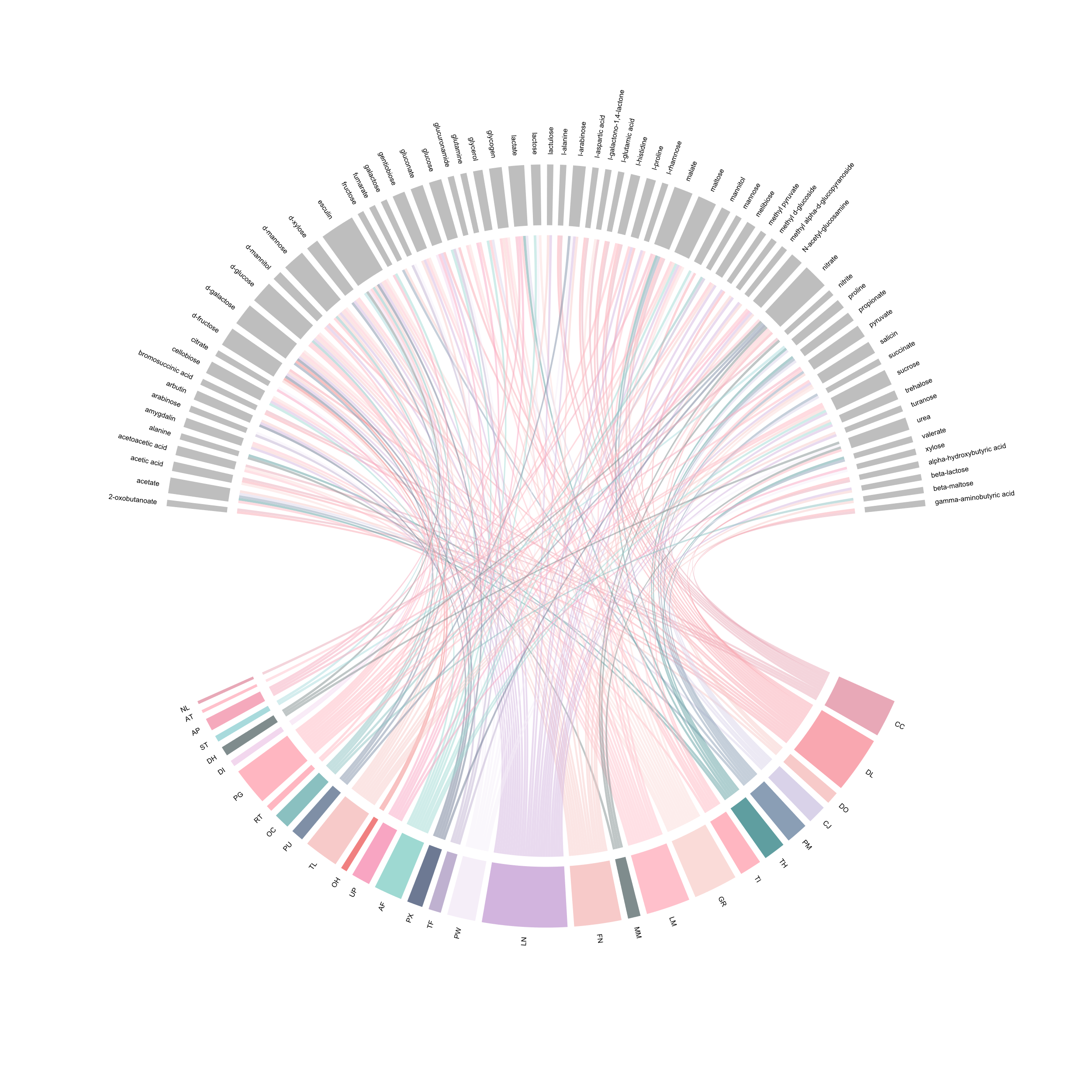
**

## Table S1. Results of 345 expanded entries based on the functional analysis.

| **Strain** | **Tax ID** | **Entry** | **New annotations** |
| --- | --- | --- | --- |
| *Nocardiopsis sp.* | 1519494 | A0A0N0TLD5 | AAA family ATPase |
| *Enterococcus durans* | 53345 | A0A5N0YZ26 | AgrB-like protein |
| *Erysipelatoclostridium ramosum* | 445974 | B0N5X1 | AgrB-like protein |
| *Eubacterium sp.* | 1262889 | R7H819 | AgrB-like protein |
| *Lachnoclostridium phytofermentans* | 357809 | A9KPE7 | AgrB-like protein |
| *Paenibacillus sp.* | 1736234 | A0A0Q4QRE0 | AgrB-like protein |
| *Paenibacillus sp.* | 1742774 | A0A329LI61 | AgrB-like protein |
| *Paenibacillus thalictri* | 2527873 | A0A4Q9DQ87 | AgrB-like protein |
| *Roseburia sp.* | 2293138 | A0A3R6KSX6 | AgrB-like protein |
| *Sellimonas intestinalis* | 1653434 | A0A3E3JYG0 | AgrB-like protein |
| *Sellimonas intestinalis* | 1653434 | A0A3E3JZ69 | AgrB-like protein |
| *Tissierella sp.* | 1280483 | A0A265QA38 | AgrB-like protein |
| *Halomonas anticariensis* | 1121939 | S2KKU1 | Autoinducer synthase |
| *Enterococcus saccharolyticus* | 1139996 | S0NGN8 | Beta-ketoacyl-[acyl-carrier-protein] synthase III C-terminal domain-containing protein |
| *Hydrogenophaga crassostreae* | 1763535 | A0A162ST26 | Beta-ketoacyl-[acyl-carrier-protein] synthase III C-terminal domain-containing protein |
| *Paenibacillus durus* | 1333534 | A0A0F7CKI0 | Beta-ketoacyl-[acyl-carrier-protein] synthase III C-terminal domain-containing protein |
| *Candidatus Contendobacter odensis* | 1400861 | A0A7U7GAX4 | CheY-like superfamily (Response regulator) |
| *Legionella drancourtii* | 658187 | G9ESD7 | CheY-like superfamily (Response regulator) |
| *Sulfuriferula nivalis* | 2675298 | A0A809S936 | CheY-like superfamily (Response regulator) |
| *unidentified eubacterium* | 50743 | A6EQ36 | CheY-like superfamily (Response regulator) |
| *Bdellovibrio bacteriovorus* | 264462 | Q6MLZ9 | Enoyl-CoA hydratase |
| *Pseudomonas sp.* | 1611770 | A0A0C5E256 | Enoyl-CoA hydratase |
| *Algoriphagus sanaruensis* | 1727163 | A0A142ERU8 | Histidine kinase |
| *Amoebophilus asiaticus* | 452471 | B3ETE3 | Histidine kinase |
| *Arsenicibacter rosenii* | 1750698 | A0A1S2VF20 | Histidine kinase |
| *Arthrobacter sp.* | 1955698 | A0A554VZ07 | Histidine kinase |
| *Bacteroides reticulotermitis* | 1445607 | W4URM0 | Histidine kinase |
| *Bacteroides sp.* | 457387 | F7M6T5 | Histidine kinase |
| *Bacteroides sp.* | 1262737 | R5J4Y6 | Histidine kinase |
| *Bacteroides sp.* | 1262737 | R5JIU3 | Histidine kinase |
| *Bacteroides sp.* | 1262740 | R7D3H3 | Histidine kinase |
| *Beggiatoa sp.* | 422289 | A7BTV4 | Histidine kinase |
| *Blautia hydrogenotrophica* | 1263061 | R5C0J1 | Histidine kinase |
| *Cloacimonas acidaminovorans* | 459349 | B0VHU9 | Histidine kinase |
| *Corynebacterium humireducens* | 1223515 | A0A0B5D9A4 | Histidine kinase |
| *Croceicoccus marinus* | 450378 | A0A1Z1FD47 | Histidine kinase |
| *Devosia enhydra* | 665118 | A0A1K2HWZ5 | Histidine kinase |
| *Ekhidna lutea* | 447679 | A0A239HXR5 | Histidine kinase |
| *Eubacterium plexicaudatum* | 1235802 | N2B4E8 | Histidine kinase |
| *Flavobacterium crassostreae* | 1763534 | A0A1B9E960 | Histidine kinase |
| *Flavobacterium sediminis* | 2201181 | A0A2U8QVZ1 | Histidine kinase |
| *Flavobacterium sediminis* | 2201181 | A0A2U8QS83 | Histidine kinase |
| *Flavobacterium sp.* | 2058314 | A0A2N1G5X0 | Histidine kinase |
| *Flavobacterium sp.* | 1929267 | A0A243SJ28 | Histidine kinase |
| *Halomonas gudaonensis* | 376427 | A0A432JIX9 | Histidine kinase |
| *Hymenobacter daecheongensis* | 1121955 | A0A1M6GU38 | Histidine kinase |
| *Hymenobacter swuensis* | 1227739 | W8ES87 | Histidine kinase |
| *Lachnospira eligens* | 515620 | C4Z237 | Histidine kinase |
| *Methylocystis heyeri* | 391905 | A0A6B8KFW2 | Histidine kinase |
| *Microterricola viridarii* | 412690 | A0A0Y0N5P4 | Histidine kinase |
| *Nitrospira sp.* | 1936990 | A0A679HMJ2 | Histidine kinase |
| *Pedobacter sp.* | 391596 | A6ED33 | Histidine kinase |
| *Polaribacter aquimarinus* | 2100726 | A0A2U2J9G4 | Histidine kinase |
| *Prevotella sp.* | 2024222 | A0A255SNY0 | Histidine kinase |
| *Rheinheimera nanhaiensis* | 562729 | I1DZ57 | Histidine kinase |
| *Rhodoplanes roseus* | 29409 | A0A327L6N7 | Histidine kinase |
| *Roseomonas deserti* | 1817963 | A0A1V2GTZ3 | Histidine kinase |
| *Runella sp.* | 2268026 | A0A3G3GJ34 | Histidine kinase |
| *Salinispira pacifica* | 1307761 | V5WJX7 | Histidine kinase |
| *Segetibacter aerophilus* | 670293 | A0A512BD76 | Histidine kinase |
| *Serinibacter arcticus* | 1655435 | A0A2U1ZV08 | Histidine kinase |
| *Sorangium cellulosum* | 448385 | A9EWU9 | Histidine kinase |
| *Sphingobacterium sp.* | 1338009 | A0A0M3CFJ8 | Histidine kinase |
| *Steroidobacter agaridevorans* | 2695856 | A0A829YDS6 | Histidine kinase |
| *Streptococcus ictaluri* | 764299 | G5K4U4 | Histidine kinase |
| *Streptococcus urinalis* | 764291 | G5KE54 | Histidine kinase |
| *Sulfuriferula nivalis* | 2675298 | A0A809RH40 | Histidine kinase |
| *Vagococcus fluvialis* | 2738 | A0A369AP97 | Histidine kinase |
| *Xanthobacter tagetidis* | 60216 | A0A3L7AEB1 | Histidine kinase |
| *Acinetobacter brisouii* | 1341683 | V2UPA8 | HTH-type transcriptional activator |
| *Acinetobacter indicus* | 1341679 | V2UD84 | HTH-type transcriptional activator |
| *Acinetobacter rudis* | 421052 | S3PP73 | HTH-type transcriptional activator |
| *Acinetobacter sp.* | 1144669 | N8W3L2 | HTH-type transcriptional activator |
| *Actinomyces glycerinitolerans* | 1892869 | A0A1M4S0U3 | HTH-type transcriptional activator |
| *Actinomyces graevenitzii* | 435830 | G9PEN9 | HTH-type transcriptional activator |
| *Actinomyces graevenitzii* | 435830 | G9PG43 | HTH-type transcriptional activator |
| *Actinomyces graevenitzii* | 435830 | G9PHT8 | HTH-type transcriptional activator |
| *Actinomyces sp.* | 888052 | E6KQF7 | HTH-type transcriptional activator |
| *Actinomyces sp.* | 653386 | G9PLZ0 | HTH-type transcriptional activator |
| *Actinomyces sp.* | 653386 | G9PMK2 | HTH-type transcriptional activator |
| *Actinomyces sp.* | 653386 | G9PPG8 | HTH-type transcriptional activator |
| *Actinomyces sp.* | 653386 | G9PPK1 | HTH-type transcriptional activator |
| *Aliiroseovarius crassostreae* | 154981 | A0A0P7J7H0 | HTH-type transcriptional activator |
| *Arthrobacter sp.* | 1494608 | A0A0A1D1X2 | HTH-type transcriptional activator |
| *Arthrobacter sp.* | 1494608 | A0A0A1D663 | HTH-type transcriptional activator |
| *Azospirillum sp.* | 716789 | A0A2U1WM00 | HTH-type transcriptional activator |
| *Bacillus sp.* | 1743142 | A0A1B8W0W4 | HTH-type transcriptional activator |
| *Bacteroides sp.* | 1262737 | R5JDV8 | HTH-type transcriptional activator |
| *Bacteroides sp.* | 1262737 | R5JEB7 | HTH-type transcriptional activator |
| *Bacteroides uniformis* | 1235787 | R9HUV1 | HTH-type transcriptional activator |
| *Bordetella sp.* | 1746199 | A0A0S1Y6G6 | HTH-type transcriptional activator |
| *Bosea sp.* | 406341 | A0A101JYM5 | HTH-type transcriptional activator |
| *Bosea sp.* | 406341 | A0A101K2F1 | HTH-type transcriptional activator |
| *Bosea sp.* | 406341 | A0A124GC63 | HTH-type transcriptional activator |
| *Caenispirillum salinarum* | 1238182 | K9HLR6 | HTH-type transcriptional activator |
| *Candidatus [Bacteroides] periocalifornicus* | 1702214 | A0A0Q4B9P0 | HTH-type transcriptional activator |
| *Candidatus Paracaedibacter acanthamoebae* | 91604 | A0A077B0V1 | HTH-type transcriptional activator |
| *Citrobacter koseri* | 290338 | A8AMP8 | HTH-type transcriptional activator |
| *Citrobacter koseri* | 290338 | A8ADL3 | HTH-type transcriptional activator |
| *Corynebacterium halotolerans* | 1121362 | M1P1I4 | HTH-type transcriptional activator |
| *Corynebacterium humireducens* | 1223515 | A0A0B5DBH2 | HTH-type transcriptional activator |
| *Corynebacterium maris* | 1224163 | S5T5K0 | HTH-type transcriptional activator |
| *Corynebacterium pyruviciproducens* | 1125779 | S2Z142 | HTH-type transcriptional activator |
| *Corynebacterium pyruviciproducens* | 1125779 | S2ZER4 | HTH-type transcriptional activator |
| *Corynebacterium sp.* | 1203562 | U7KZ14 | HTH-type transcriptional activator |
| *Desulfosarcina widdelii* | 947919 | A0A5K7Z0Y9 | HTH-type transcriptional activator |
| *Desulfovibrio sp.* | 665942 | G1UR80 | HTH-type transcriptional activator |
| *Dialister sp.* | 1262869 | R7CQL2 | HTH-type transcriptional activator |
| *Dialister succinatiphilus* | 742743 | H1D263 | HTH-type transcriptional activator |
| *Enterococcus aquimarinus* | 328396 | A0A1L8QWE0 | HTH-type transcriptional activator |
| *Enterococcus phoeniculicola* | 1158610 | R3W9C1 | HTH-type transcriptional activator |
| *Enterococcus ratti* | 150033 | A0A1L8WRR2 | HTH-type transcriptional activator |
| *Enterococcus ratti* | 150033 | A0A1L8WSL6 | HTH-type transcriptional activator |
| *Exiguobacterium chiriqhucha* | 1345023 | U1N551 | HTH-type transcriptional activator |
| *Facklamia hominis* | 883111 | K1MJP9 | HTH-type transcriptional activator |
| *Facklamia languida* | 883113 | H3NGX7 | HTH-type transcriptional activator |
| *Flavobacterium tangerinum* | 2488728 | A0A3P3W727 | HTH-type transcriptional activator |
| *Fusobacterium necrophorum* | 742814 | H1DC52 | HTH-type transcriptional activator |
| *Gemmata massiliana* | 1210884 | A0A6P2D200 | HTH-type transcriptional activator |
| *Granulicatella elegans* | 626369 | D0BKY8 | HTH-type transcriptional activator |
| *Hymenobacter sp.* | 1356852 | A0A076HYK4 | HTH-type transcriptional activator |
| *Hymenobacter swuensis* | 1227739 | W8EY71 | HTH-type transcriptional activator |
| *Hymenobacter swuensis* | 1227739 | W8EYL7 | HTH-type transcriptional activator |
| *Hymenobacter swuensis* | 1227739 | W8F0K1 | HTH-type transcriptional activator |
| *Hymenobacter swuensis* | 1227739 | W8F5U1 | HTH-type transcriptional activator |
| *Hymenobacter swuensis* | 1227739 | W8F8T9 | HTH-type transcriptional activator |
| *Hymenobacter swuensis* | 1227739 | W8F3N5 | HTH-type transcriptional activator |
| *Hymenobacter swuensis* | 1227739 | W8F5D5 | HTH-type transcriptional activator |
| *Janibacter indicus* | 857417 | A0A1L3MD86 | HTH-type transcriptional activator |
| *Kribbella turkmenica* | 2530375 | A0A4R4WZ73 | HTH-type transcriptional activator |
| *Lactiplantibacillus mudanjiangensis* | 1296538 | A0A660E0A3 | HTH-type transcriptional activator |
| *Lactococcus reticulitermitis* | 2025039 | A0A224X9E8 | HTH-type transcriptional activator |
| *Leeuwenhoekiella blandensis* | 398720 | A3XHS2 | HTH-type transcriptional activator |
| *Leeuwenhoekiella blandensis* | 398720 | A3XN23 | HTH-type transcriptional activator |
| *Leeuwenhoekiella blandensis* | 398720 | A3XPB6 | HTH-type transcriptional activator |
| *Legionella drancourtii* | 658187 | G9EJH0 | HTH-type transcriptional activator |
| *Levilinea saccharolytica* | 229921 | A0A0P6YTX9 | HTH-type transcriptional activator |
| *Loigolactobacillus rennini* | 1423796 | A0A0R2D4G8 | HTH-type transcriptional activator |
| *Marinoscillum sp.* | 2653151 | A0A654E139 | HTH-type transcriptional activator |
| *Massilia sp.* | 1549812 | A0A086W8F4 | HTH-type transcriptional activator |
| *Massilia sp.* | 1406431 | A0A0J1DAC1 | HTH-type transcriptional activator |
| *Massilia timonae* | 883126 | K9D5N5 | HTH-type transcriptional activator |
| *Massilia timonae* | 883126 | K9D965 | HTH-type transcriptional activator |
| *Massilia timonae* | 883126 | K9DDG3 | HTH-type transcriptional activator |
| *Massilia timonae* | 883126 | K9DJP5 | HTH-type transcriptional activator |
| *Massilia timonae* | 883126 | K9DQD5 | HTH-type transcriptional activator |
| *Massilia timonae* | 883126 | K9E5G6 | HTH-type transcriptional activator |
| *Methyloglobulus morosus* | 1116472 | V5BDL9 | HTH-type transcriptional activator |
| *Microbacterium sp.* | 367477 | A0A0U4H640 | HTH-type transcriptional activator |
| *Microvirga vignae* | 1225564 | A0A0H1RI99 | HTH-type transcriptional activator |
| *Mucilaginibacter polytrichastri* | 1302689 | A0A1Q5ZXM2 | HTH-type transcriptional activator |
| *Mucilaginibacter polytrichastri* | 1302689 | A0A1Q6A391 | HTH-type transcriptional activator |
| *Mucilaginibacter polytrichastri* | 1302689 | A0A1Q6A3S5 | HTH-type transcriptional activator |
| *Mucilaginibacter polytrichastri* | 1302689 | A0A1Q5ZXG1 | HTH-type transcriptional activator |
| *Mucilaginibacter polytrichastri* | 1302689 | A0A1Q6A0A2 | HTH-type transcriptional activator |
| *Mucilaginibacter polytrichastri* | 1302689 | A0A1Q6A245 | HTH-type transcriptional activator |
| *Mucilaginibacter polytrichastri* | 1302689 | A0A1Q6A439 | HTH-type transcriptional activator |
| *Mucilaginibacter polytrichastri* | 1302689 | A0A1Q6A5H6 | HTH-type transcriptional activator |
| *Mucilaginibacter polytrichastri* | 1302689 | A0A1Q6A603 | HTH-type transcriptional activator |
| *Nocardioides sp.* | 2653157 | A0A653YUZ7 | HTH-type transcriptional activator |
| *Nocardioides sp.* | 2653157 | A0A653ZS42 | HTH-type transcriptional activator |
| *Nocardioides sp.* | 2662361 | A0A5Q0NQ66 | HTH-type transcriptional activator |
| *Nocardioides sp.* | 2662361 | A0A5Q0NSD2 | HTH-type transcriptional activator |
| *Nocardioides sp.* | 2662361 | A0A5Q0NY67 | HTH-type transcriptional activator |
| *Novosphingobium guangzhouense* | 1850347 | A0A2K2G3G3 | HTH-type transcriptional activator |
| *Oleiphilus sp.* | 1822254 | A0A657BAZ1 | HTH-type transcriptional activator |
| *Paenibacillus athensensis* | 1967502 | A0A4Y8PPL4 | HTH-type transcriptional activator |
| *Paenibacillus athensensis* | 1967502 | A0A4Y8PSD2 | HTH-type transcriptional activator |
| *Paenibacillus athensensis* | 1967502 | A0A4Y8PVC6 | HTH-type transcriptional activator |
| *Paenibacillus athensensis* | 1967502 | A0A4Y8PW50 | HTH-type transcriptional activator |
| *Paenibacillus contaminans* | 450362 | A0A329MMQ2 | HTH-type transcriptional activator |
| *Paenibacillus nuruki* | 1886670 | A0A1E3L5G0 | HTH-type transcriptional activator |
| *Paenibacillus pectinilyticus* | 512399 | A0A1C1A743 | HTH-type transcriptional activator |
| *Paenibacillus rigui* | 554312 | A0A229UGT2 | HTH-type transcriptional activator |
| *Paenibacillus riograndensis* | 1073571 | A0A0E4H859 | HTH-type transcriptional activator |
| *Paenibacillus sp.* | 1566358 | A0A0D3V7H1 | HTH-type transcriptional activator |
| *Paraglaciecola arctica* | 493475 | K6XNL8 | HTH-type transcriptional activator |
| *Paraglaciecola arctica* | 493475 | K6Z7F9 | HTH-type transcriptional activator |
| *Paraglaciecola psychrophila* | 1129794 | K7A3H3 | HTH-type transcriptional activator |
| *Pedobacter sp.* | 1736297 | A0A0Q5TME2 | HTH-type transcriptional activator |
| *Pedobacter sp.* | 509635 | W6TZ22 | HTH-type transcriptional activator |
| *Persicitalea jodogahamensis* | 402147 | A0A8J3D5J6 | HTH-type transcriptional activator |
| *Phaeodactylibacter xiamenensis* | 1524460 | A0A098S2K0 | HTH-type transcriptional activator |
| *Planktothrix agardhii* | 388467 | A0A073CDG5 | HTH-type transcriptional activator |
| *Planktothrix agardhii* | 388467 | A0A073CEM8 | HTH-type transcriptional activator |
| *Planktothrix agardhii* | 388467 | A0A073CM41 | HTH-type transcriptional activator |
| *Prevotella sp.* | 1262930 | R6A3V2 | HTH-type transcriptional activator |
| *Prevotella sp.* | 1262934 | R6XF68 | HTH-type transcriptional activator |
| *Prevotella sp.* | 1262937 | R7LD29 | HTH-type transcriptional activator |
| *Propionibacterium sp.* | 1203605 | S3X8F8 | HTH-type transcriptional activator |
| *Propionibacterium sp.* | 1203605 | S3XAL6 | HTH-type transcriptional activator |
| *Propionibacterium sp.* | 1203605 | S3XBK9 | HTH-type transcriptional activator |
| *Propionibacterium sp.* | 1203605 | S3XJI4 | HTH-type transcriptional activator |
| *Propionibacterium sp.* | 1203605 | S3XL86 | HTH-type transcriptional activator |
| *Propionibacterium sp.* | 1203605 | S3XN08 | HTH-type transcriptional activator |
| *Propionimicrobium lymphophilum* | 883161 | S2WMU9 | HTH-type transcriptional activator |
| *Pseudomonas corrugata* | 47879 | A0A3M3EC83 | HTH-type transcriptional activator |
| *Pseudomonas corrugata* | 47879 | A0A3M3EUI1 | HTH-type transcriptional activator |
| *Pseudomonas sp.* | 1203578 | N2JD87 | HTH-type transcriptional activator |
| *Pseudomonas sp.* | 1611770 | A0A0C5DY98 | HTH-type transcriptional activator |
| *Pseudomonas sp.* | 1611770 | A0A0C5E3W9 | HTH-type transcriptional activator |
| *Pseudomonas sp.* | 1611770 | A0A0C5E5C1 | HTH-type transcriptional activator |
| *Pseudomonas sp.* | 1611770 | A0A0C5EAE3 | HTH-type transcriptional activator |
| *Pseudomonas sp.* | 1611770 | A0A0C5ED80 | HTH-type transcriptional activator |
| *Pseudomonas sp.* | 1611770 | A0A0C5EP46 | HTH-type transcriptional activator |
| *Ramlibacter tataouinensis* | 94132 | A0A127JWL2 | HTH-type transcriptional activator |
| *Ramlibacter tataouinensis* | 94132 | A0A127K040 | HTH-type transcriptional activator |
| *Rhodovulum sp.* | 1187851 | J9DEB4 | HTH-type transcriptional activator |
| *Rothia aeria* | 172042 | A0A2Z5QVD0 | HTH-type transcriptional activator |
| *Rothia kristinae* | 37923 | A0A199NSZ7 | HTH-type transcriptional activator |
| *Siphonobacter sp.* | 1864822 | A0A261PZN8 | HTH-type transcriptional activator |
| *Sphingobacterium soli* | 1914757 | A0A8H9KT02 | HTH-type transcriptional activator |
| *Sphingobium chungbukense* | 56193 | A0A0M3ATX7 | HTH-type transcriptional activator |
| *Sphingobium sp.* | 2082188 | A0A2Z6AIS6 | HTH-type transcriptional activator |
| *Sphingobium yanoikuyae* | 883163 | K9CVX2 | HTH-type transcriptional activator |
| *Sphingomonas sp.* | 2653171 | A0A654CE08 | HTH-type transcriptional activator |
| *Sporocytophaga myxococcoides* | 153721 | A0A098LGB0 | HTH-type transcriptional activator |
| *Sutterella sp.* | 1262976 | R7BYV1 | HTH-type transcriptional activator |
| *Sutterella wadsworthensis* | 742823 | K1KJ12 | HTH-type transcriptional activator |
| *Sutterella wadsworthensis* | 1203554 | S3B8D0 | HTH-type transcriptional activator |
| *Sutterella wadsworthensis* | 1203554 | S3BES4 | HTH-type transcriptional activator |
| *Tenacibaculum sp.* | 754426 | A0A2S7TIS1 | HTH-type transcriptional activator |
| *Tetrasphaera japonica* | 1194083 | A0A077LU01 | HTH-type transcriptional activator |
| *Thiobacillus denitrificans* | 36861 | A0A106BJN9 | HTH-type transcriptional activator |
| *Tuwongella immobilis* | 692036 | A0A6C2YPJ3 | HTH-type transcriptional activator |
| *Vibrio campbellii* | 2902295 | A7N5G9 | HTH-type transcriptional activator |
| *Lewinella sp.* | 1803372 | A0A180EMI6 | Peptidase M4 family protein |
| *Nocardioides sp.* | 1027620 | A0A401Y283 | Peptidase M4 family protein |
| *Nocardioides sp.* | 1736488 | A0A0Q8PVL3 | Peptidase M4 family protein |
| *Paucibacter sp.* | 1768242 | A0A0U2LUF3 | Peptidase M4 family protein |
| *Rubrivirga marina* | 1196024 | A0A271J3L8 | Peptidase M4 family protein |
| *Rubrivirga marina* | 1196024 | A0A271J5K6 | Peptidase_M4 |
| *Aeromicrobium chenweiae* | 2079793 | A0A2S0WJQ6 | Response regulator |
| *Aeromicrobium chenweiae* | 2079793 | A0A2S0WMN6 | Response regulator |
| *Arthrobacter psychrochitiniphilus* | 291045 | A0A2V3DVF7 | Response regulator |
| *Blastopirellula retiformator* | 2527970 | A0A5C5VL92 | Response regulator |
| *Candidatus Nitrotoga sp.* | 2559597 | A0A455XE65 | Response regulator |
| *Flavobacterium rivuli* | 1121895 | A0A0A2M3A6 | Response regulator |
| *Hydrogenophaga crassostreae* | 1763535 | A0A162VRA1 | Response regulator |
| *Janthinobacterium sp.* | 375286 | A6SZF4 | Response regulator |
| *Keratinibaculum paraultunense* | 1278232 | A0A4R3KXT7 | Response regulator |
| *Mucilaginibacter phyllosphaerae* | 1812349 | A0A4Y8AC13 | Response regulator |
| *Mucilaginibacter phyllosphaerae* | 1812349 | A0A4Y8AIB5 | Response regulator |
| *Oleiphilus sp.* | 1822233 | A0A657ANK1 | Response regulator |
| *Oleiphilus sp.* | 1822242 | A0A657B0P9 | Response regulator |
| *Oleiphilus sp.* | 1822242 | A0A657B4Q3 | Response regulator |
| *Oleiphilus sp.* | 1822254 | A0A657B5N9 | Response regulator |
| *Oleiphilus sp.* | 1822254 | A0A657BCF6 | Response regulator |
| *Paenibacillus cremeus* | 2163881 | A0A559K438 | Response regulator |
| *Planktothrix tepida* | 671072 | A0A1J1LHX9 | Response regulator |
| *Rhodopirellula pilleata* | 2714738 | A0A5C5ZY22 | Response regulator |
| *Segetibacter aerophilus* | 670293 | A0A512BGX4 | Response regulator |
| *Sphingomonas melonis* | 621456 | A0A175Y041 | Response regulator |
| *Tessaracoccus lapidicaptus* | 1427523 | A0A1C0AMC1 | Response regulator |
| *Thiohalocapsa sp.* | 1385625 | V4JB07 | Response regulator |
| *Tissierella praeacuta* | 1123404 | A0A1M4TFF3 | Response regulator |
| *Candidatus Berkiella aquae* | 295108 | A0A0Q9YK95 | Response regulator transcription factor |
| *Legionella feeleii* | 453 | A0A378IW37 | Response regulator transcription factor |
| *Massilia forsythiae* | 2728020 | A0A7Z2VX20 | Response regulator transcription factor |
| *Mesorhizobium amorphae* | 1449348 | A0A212DKY0 | Response regulator transcription factor |
| *Paracoccus marcusii* | 59779 | A0A843Y4S0 | Response regulator transcription factor |
| *Paracoccus marcusii* | 59779 | A0A843Y4Z5 | Response regulator transcription factor |
| *Candidatus Manganitrophus noduliformans* | 2606439 | A0A7X6ICU4 | Thiolase-like protein type 1 additional C-terminal domain-containing protein |
| *Prevotella histicola* | 857291 | G6AIZ6 | Thiolase-like protein type 1 additional C-terminal domain-containing protein |
| *Peptostreptococcaceae bacterium* | 1321784 | U2JX77 | Transcription activator |
| *Arthrobacter psychrochitiniphilus* | 291045 | A0A2V3DVS0 | Transcriptional activator |
| *Arthrobacter psychrochitiniphilus* | 291045 | A0A2V3DWD5 | Transcriptional activator |
| *Arthrobacter psychrochitiniphilus* | 291045 | A0A2V3DWN8 | Transcriptional activator |
| *Bacteroides sp.* | 1262737 | R5JPX4 | Transcriptional activator |
| *Gemmata massiliana* | 1210884 | A0A6P2D0H6 | Transcriptional activator |
| *Gemmata massiliana* | 1210884 | A0A6P2D4J5 | Transcriptional activator |
| *Niallia circulans* | 1397 | A0A0J1LCX4 | Transcriptional activator |
| *Pseudomonas agarici* | 46677 | A0A0X1T6N0 | Transcriptional activator |
| *Pseudomonas agarici* | 46677 | A0A0X1T6Q8 | Transcriptional activator |
| *Pseudomonas sp.* | 1203578 | N2JKD8 | Transcriptional activator |
| *Tatlockia micdadei* | 451 | A0A098GH61 | Transcriptional activator |
| *Vagococcus fluvialis* | 2738 | A0A369AWN1 | Transcriptional activator |
| *Legionella antarctica* | 2708020 | A0A6F8T3F3 | Transcriptional activator TraM |
| *Legionella antarctica* | 2708020 | A0A6F8T598 | Transcriptional activator TraM |
| *Acetobacter sp.* | 1670658 | A0A1Y3FYE5 | Two component response regulator of OmpR/PhoB family protein |
| *Acinetobacter brisouii* | 1341683 | V2UR18 | Two component response regulator of OmpR/PhoB family protein |
| *Acinetobacter gerneri* | 1120926 | N8ZMZ1 | Two component response regulator of OmpR/PhoB family protein |
| *Acinetobacter guillouiae* | 1217656 | N8WTE9 | Two component response regulator of OmpR/PhoB family protein |
| *Acinetobacter guillouiae* | 1217656 | N8Y7A9 | Two component response regulator of OmpR/PhoB family protein |
| *Acinetobacter guillouiae* | 1217656 | N8Y827 | Two component response regulator of OmpR/PhoB family protein |
| *Acinetobacter sp.* | 1144669 | N8XGJ1 | Two component response regulator of OmpR/PhoB family protein |
| *Acinetobacter sp.* | 1144669 | N8XHQ5 | Two component response regulator of OmpR/PhoB family protein |
| *Actinomyces sp.* | 653386 | G9PMI8 | Two component response regulator of OmpR/PhoB family protein |
| *Alistipes indistinctus* | 742725 | G5HA43 | Two component response regulator of OmpR/PhoB family protein |
| *Atopobium minutum* | 997872 | N2BP61 | Two component response regulator of OmpR/PhoB family protein |
| *Bacteroides cellulosilyticus* | 1263038 | R6KEV4 | Two component response regulator of OmpR/PhoB family protein |
| *Bacteroides cellulosilyticus* | 1263038 | R6KJH7 | Two component response regulator of OmpR/PhoB family protein |
| *Bacteroides plebeius* | 1263052 | R5W1G3 | Two component response regulator of OmpR/PhoB family protein |
| *Bacteroides sp.* | 457387 | F7LXS5 | Two component response regulator of OmpR/PhoB family protein |
| *Bacteroides sp.* | 457387 | F7LYS6 | Two component response regulator of OmpR/PhoB family protein |
| *Bacteroides sp.* | 1262737 | R5J9P2 | Two component response regulator of OmpR/PhoB family protein |
| *Bacteroides sp.* | 1262751 | R6T7C6 | Two component response regulator of OmpR/PhoB family protein |
| *Ciceribacter selenitireducens* | 1336235 | A0A376AK23 | Two component response regulator of OmpR/PhoB family protein |
| *Cronobacter sakazakii* | 290339 | A7MGC7 | Two component response regulator of OmpR/PhoB family protein |
| *Dialister succinatiphilus* | 742743 | H1CXP5 | Two component response regulator of OmpR/PhoB family protein |
| *Dysgonomonas gadei* | 742766 | F5IXA4 | Two component response regulator of OmpR/PhoB family protein |
| *Dysgonomonas gadei* | 742766 | F5J143 | Two component response regulator of OmpR/PhoB family protein |
| *Dysgonomonas gadei* | 742766 | F5J279 | Two component response regulator of OmpR/PhoB family protein |
| *Dysgonomonas mossii* | 742767 | F8X1P7 | Two component response regulator of OmpR/PhoB family protein |
| *Dysgonomonas mossii* | 742767 | F8X4B7 | Two component response regulator of OmpR/PhoB family protein |
| *Enterococcus canis* | 214095 | A0A1L8RCU3 | Two component response regulator of OmpR/PhoB family protein |
| *Gemmata massiliana* | 1210884 | A0A6P2DI30 | Two component response regulator of OmpR/PhoB family protein |
| *Granulicatella elegans* | 626369 | D0BNB3 | Two component response regulator of OmpR/PhoB family protein |
| *Helicobacter bilis* | 613026 | C3XEK1 | Two component response regulator of OmpR/PhoB family protein |
| *Helicobacter bilis* | 613026 | C3XI59 | Two component response regulator of OmpR/PhoB family protein |
| *Helicobacter macacae* | 1357400 | V8CDT8 | Two component response regulator of OmpR/PhoB family protein |
| *Lactococcus reticulitermitis* | 2025039 | A0A224X9G8 | Two component response regulator of OmpR/PhoB family protein |
| *Lactococcus reticulitermitis* | 2025039 | A0A224XBG7 | Two component response regulator of OmpR/PhoB family protein |
| *Leuconostoc fallax* | 1251 | A0A4R5N9E5 | Two component response regulator of OmpR/PhoB family protein |
| *Leuconostoc fallax* | 1251 | A0A4R5NC07 | Two component response regulator of OmpR/PhoB family protein |
| *Levilinea saccharolytica* | 229921 | A0A0P6YNH8 | Two component response regulator of OmpR/PhoB family protein |
| *Luteimonas sp.* | 1547516 | A0A0H1AEV5 | Two component response regulator of OmpR/PhoB family protein |
| *Lysobacter sp.* | 1736612 | A0A0Q9EA77 | Two component response regulator of OmpR/PhoB family protein |
| *Massilia timonae* | 883126 | K9DFC4 | Two component response regulator of OmpR/PhoB family protein |
| *Massilia timonae* | 883126 | K9DFH4 | Two component response regulator of OmpR/PhoB family protein |
| *Massilia timonae* | 883126 | K9DG85 | Two component response regulator of OmpR/PhoB family protein |
| *Mycolicibacterium paratuberculosis* | 262316 | Q741T5 | Two component response regulator of OmpR/PhoB family protein |
| *Ornatilinea apprima* | 1134406 | A0A0P6Y863 | Two component response regulator of OmpR/PhoB family protein |
| *Paenibacillus riograndensis* | 1073571 | A0A0E4CUV5 | Two component response regulator of OmpR/PhoB family protein |
| *Paenibacillus sp.* | 1499968 | A0A069D9T8 | Two component response regulator of OmpR/PhoB family protein |
| *Planktothrix agardhii* | 388467 | A0A073CMP0 | Two component response regulator of OmpR/PhoB family protein |
| *Propionibacterium sp.* | 1203605 | S3XD61 | Two component response regulator of OmpR/PhoB family protein |
| *Propionibacterium sp.* | 1203605 | S3YEC1 | Two component response regulator of OmpR/PhoB family protein |
| *Pseudohongiella spirulinae* | 1249552 | A0A0S2KFZ9 | Two component response regulator of OmpR/PhoB family protein |
| *Pseudomonas corrugata* | 47879 | A0A3M3E104 | Two component response regulator of OmpR/PhoB family protein |
| *Pseudomonas corrugata* | 47879 | A0A3M3ER38 | Two component response regulator of OmpR/PhoB family protein |
| *Pseudomonas corrugata* | 47879 | A0A3M3EU25 | Two component response regulator of OmpR/PhoB family protein |
| *Pseudomonas sp.* | 1203578 | N2JBC6 | Two component response regulator of OmpR/PhoB family protein |
| *Pseudomonas sp.* | 1611770 | A0A0C5DUM4 | Two component response regulator of OmpR/PhoB family protein |
| *Pseudomonas sp.* | 1611770 | A0A0C5EEH1 | Two component response regulator of OmpR/PhoB family protein |
| *Pseudomonas sp.* | 1611770 | A0A0C5EFA4 | Two component response regulator of OmpR/PhoB family protein |
| *Pseudonocardia sp.* | 1641402 | A0A0M4Q6R5 | Two component response regulator of OmpR/PhoB family protein |
| *Salmonella arizonae* | 41514 | A9MQI3 | Two component response regulator of OmpR/PhoB family protein |
| *Sphingobium yanoikuyae* | 883163 | K9DAZ7 | Two component response regulator of OmpR/PhoB family protein |
| *Sphingobium yanoikuyae* | 883163 | K9DEM8 | Two component response regulator of OmpR/PhoB family protein |
| *Stenotrophomonas daejeonensis* | 659018 | A0A0R0DS31 | Two component response regulator of OmpR/PhoB family protein |
| *Sutterella wadsworthensis* | 1203554 | S3BE16 | Two component response regulator of OmpR/PhoB family protein |
| *Thermanaerothrix daxensis* | 869279 | A0A0P6XS11 | Two component response regulator of OmpR/PhoB family protein |
| *Tuwongella immobilis* | 692036 | A0A6C2YMU9 | Two component response regulator of OmpR/PhoB family protein |
| *Weissella kandleri* | 1616 | A0A0R2JBL1 | Two component response regulator of OmpR/PhoB family protein |

## Table S2. Average relative abundance and distribution of activated sludge core bacterial communities.

|  | **phylum** | **genera** | **species** | **abundance (distribution)** |
| --- | --- | --- | --- | --- |
| 1 | Pseudomonadota | Dokdonella | *Dokdonella immobilis* | 1.52% (90.12%) |
| 2 | Bacteroidota | Lacibacter | *Lacibacter nakdongensis* | 1.21% (84.58%) |
| 3 | Bacteroidota | Phaeodactylibacter | *Phaeodactylibacter xiamenensis* | 1.08% (87.75%) |
| 4 | Bacteroidota | Flavilitoribacter | *Flavilitoribacter nigricans* | 0.99% (90.51%) |
| 5 | Bacteroidota | Owenweeksia | *Owenweeksia hongkongensis* | 0.98% (90.12%) |
| 6 | Nitrospirota | Nitrospira | *Nitrospira lenta* | 0.71% (98.02%) |
| 7 | Bacteroidota | Terrimonas | *Terrimonas lutea* | 0.71% (96.05%) |
| 8 | Bacteroidota | Phaeodactylibacter | *Phaeodactylibacter xiamenensis* | 0.69% (85.38%) |
| 9 | Bacteroidota | Geofilum | *Geofilum rhodophaeum* | 0.54% (88.54%) |
| 10 | Pseudomonadota | Thauera | *Thauera humireducens* | 0.52% (98.81%) |
| 11 | Pseudomonadota | Thiocapsa | *Thiocapsa imhoffii* | 0.51% (82.21%) |
| 12 | Ignavibacteriota | Ignavibacterium | *Ignavibacterium album* | 0.49% (94.86%) |
| 13 | Pseudomonadota | Povalibacter | *Povalibacter uvarum* | 0.45% (81.82%) |
| 14 | Bacteroidota | Flavilitoribacter | *Flavilitoribacter nigricans* | 0.45% (80.24%) |
| 15 | Pseudomonadota | Allochromatium | *Allochromatium palmeri* | 0.44% (88.14%) |
| 16 | Pseudomonadota | Usitatibacter | *Usitatibacter palustris* | 0.42% (87.35%) |
| 17 | Actinomycetota | Actinomarinicola | *Actinomarinicola tropica* | 0.41% (94.47%) |
| 18 | Bacteroidota | Geofilum | *Geofilum rhodophaeum* | 0.40% (87.75%) |
| 19 | Myxococcota | Haliangium | *Haliangium ochraceum* | 0.40% (87.35%) |
| 20 | Pseudomonadota | Dechloromonas | *Dechloromonas hortensis* | 0.38% (98.02%) |
| 21 | Bacteroidota | Pseudobacter | *Pseudobacter ginsenosidimutans* | 0.35% (98.42%) |
| 22 | Pseudomonadota | Crenobacter | *Crenobacter cavernae* | 0.35% (98.02%) |
| 23 | Pseudomonadota | Paracidovorax | *Paracidovorax wautersii* | 0.35% (100.00%) |
| 24 | Pseudomonadota | Acidibacter | *Acidibacter ferrireducens* | 0.34% (96.44%) |
| 25 | Bacteroidota | Flavilitoribacter | *Flavilitoribacter nigricans* | 0.34% (80.63%) |
| 26 | Pseudomonadota | Ahniella | *Ahniella affigens* | 0.33% (91.70%) |
| 27 | Acidobacteriota | Stenotrophobacter | *Stenotrophobacter terrae* | 0.32% (85.77%) |
| 28 | Pseudomonadota | Comamonas | *Comamonas jiangduensis* | 0.32% (100.00%) |
| 29 | Bacteroidota | Labilibacter | *Labilibacter marinus* | 0.31% (95.65%) |
| 30 | Pseudomonadota | Propionivibrio | *Propionivibrio militaris* | 0.31% (86.96%) |
| 31 | Bacillota | Romboutsia | *Romboutsia timonensis* | 0.28% (100.00%) |
| 32 | Bacteroidota | Cloacibacterium | *Cloacibacterium caeni* | 0.25% (100.00%) |
| 33 | Pseudomonadota | Delftia | *Delftia litopenaei* | 0.24% (99.60%) |
| 34 | Pseudomonadota | Aquisediminimonas | *Aquisediminimonas profunda* | 0.24% (97.23%) |
| 35 | Pseudomonadota | Dechloromonas | *Dechloromonas hortensis* | 0.24% (84.98%) |
| 36 | Pseudomonadota | Denitratisoma | *Denitratisoma oestradiolicum* | 0.23% (84.19%) |
| 37 | Chloroflexota | Tepidiforma | *Tepidiforma flava* | 0.21% (94.47%) |
| 38 | Pseudomonadota | Woodsholea | *Woodsholea maritima* | 0.21% (91.30%) |
| 39 | Bacteroidota | Prolixibacter | *Prolixibacter bellariivorans* | 0.21% (90.51%) |
| 40 | Pseudomonadota | Methyloglobulus | *Methyloglobulus morosus* | 0.21% (88.14%) |

## References

1. Chen Z, Zhao P, Li F, Leier A, Marquez-Lago TT, Wang Y, et al. *iFeature: a Python package and web server for features extraction and selection from protein and peptide sequences.* Bioinformatics. 2018; **34**: 2499-2502. <https://doi.org/10.1093/bioinformatics/bty140>.

2. Cortes C, Vapnik V. *Support-vector networks.* Machine Learning. 1995; **20**: 273-297. <https://doi.org/10.1007/BF00994018>.

3. Peterson LE. *K-nearest neighbor.* Scholarpedia. 2009; **4**: 1883.

4. Breiman L. *Random Forests.* Machine Learning. 2001; **45**: 5-32. <https://doi.org/10.1023/A:1010933404324>.

5. LeCun Y, Bengio Y, Hinton G. *Deep learning.* Nature. 2015; **521**: 436-444. <https://doi.org/10.1038/nature14539>.

6. Pedregosa F, Varoquaux, G., Gramfort, A., Michel, V., Thirion, B., Grisel, O., Blondel, M., Prettenhofer, P., Weiss, R., Dubourg, V., Vanderplas, J., Passos, A., Cournapeau, D., Brucher, M., Perrot, M., Duchesnay, E. *Scikit-learn: Machine Learning in Python.* Journal of Machine Learning Research. 2011; **12**: 2825-2830.

7. Agarap AF. *Deep Learning using Rectified Linear Units (ReLU).* ArXiv. 2018; **abs/1803.08375**.
